# Marine siliceous ecosystem decline led to sustained anomalous Early Triassic warmth

**DOI:** 10.1038/s41467-022-31128-3

**Published:** 2022-06-18

**Authors:** Terry T. Isson, Shuang Zhang, Kimberly V. Lau, Sofia Rauzi, Nicholas J. Tosca, Donald E. Penman, Noah J. Planavsky

**Affiliations:** 1 Te Aka Mātuatua, University of Waikato (Tauranga), BOP, Tauranga, New Zealand; 2 Department of Oceanography, Texas A&M University, College Station, TX USA; 3 Department of Geosciences and Earth and Environmental Systems Institute, Penn State University, University Park, PA USA; 4 Department of Earth Sciences, University of Cambridge, Cambridge, UK; 5 Department of Geosciences, Utah State University, Logan, UT USA; 6 Department of Earth and Planetary Sciences, Yale University, New Haven, CT USA

**Keywords:** Palaeoclimate, Carbon cycle

## Abstract

In the wake of rapid CO_2_ release tied to the emplacement of the Siberian Traps, elevated temperatures were maintained for over five million years during the end-Permian biotic crisis. This protracted recovery defies our current understanding of climate regulation via the silicate weathering feedback, and hints at a fundamentally altered carbon and silica cycle. Here, we propose that the development of widespread marine anoxia and Si-rich conditions, linked to the collapse of the biological silica factory, warming, and increased weathering, was capable of trapping Earth’s system within a hyperthermal by enhancing ocean-atmosphere CO_2_ recycling via authigenic clay formation. While solid-Earth degassing may have acted as a trigger, subsequent biotic feedbacks likely exacerbated and prolonged the environmental crisis. This refined view of the carbon-silica cycle highlights that the ecological success of siliceous organisms exerts a potentially significant influence on Earth’s climate regime.

## Abstract (web summary)

The widespread disappearance of siliceous life sustained extreme temperatures in the wake of Earth’s most severe mass extinction event.

## Introduction

Global warming at the end-Permian initiated the most adverse and extended environmental crisis in the Phanerozoic^1–5^. This is the only known climate perturbation where carbon release rates and the initial pace of warming may have been comparable to modern rates^6–9^. A rapid temperature rise of >10 °C^2,10^, acidification^11^, and marine O_2_ decline^12,13^ drove a massive loss in biodiversity and a major shift in marine ecosystem structure^5^. An estimated 85–95% of all marine animal species went extinct across the latest Permian/Early Triassic transition—making it the most severe extinction event in Earth’s history^1,5,13^. Despite the growing consensus for a causal link between the establishment of warm and anoxic oceans and the widespread loss of marine biodiversity^4,12,13^, basic aspects of the end-Permian crisis remain enigmatic. Of particular interest are the records of extreme temperature that cannot be easily explained with the standard view of climate regulation during hyperthermals.

**Fig. 1:**
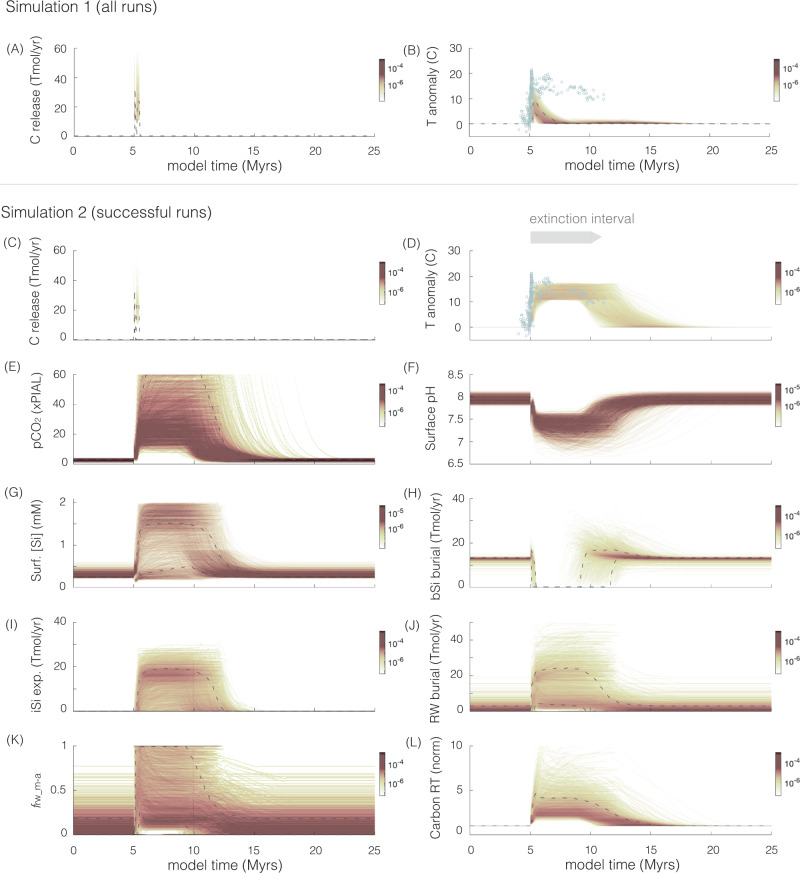
Simulation 1 (degassing + fixed carbon recycling) results (*n* = 10,000) and successful model results from Simulation 2 (degassing + dynamic carbon recycling). Color bars indicate the frequency (normalized) of the results, 68% of the values are within the dashed lines. Model time of 5 Myrs marks the initiation of volcanic carbon release and the onset of extinction. Range of parameters explored: carbon release = 30,000–55,000 Pg; release duration = 0.8 × 10^5^–0.24 × 10^6^ years; and climate sensitivity = 2–5 °C (Supplementary Tables 2 and 3). **A**, **C** carbon released from solid earth (volcanic) and sedimentary metamorphic degassing fluxes (Tmol/yr); **B**, **D** temperature anomaly, compared to sea surface temperature reconstructions (blue circles)^2,32,33^ with age constraints based on refs. ^9,87^; **E** *p*CO_2_ (times preindustrial atmospheric level (×PIAL)); **F** surface pH; **G** surface-dissolved Si (mM); and **H** biogenic, **I** inorganic, and **J** authigenic clay silica export fluxes (Tmol/yr); **K** *f*_rw_m-a_; **L** residence time of carbon normalized to the background value. Unfiltered results (*n* = 10,000) for Simulation 2 are presented in Supplementary Figs. 6 and 7.

Long-term climate on Earth is regulated by the coupled global carbon and silica cycles^14–22^. The neutralization of CO_2_ through the weathering of primary silicate materials, when tied to the precipitation of carbonate (CaCO_3_) and chert (SiO_2_), sequesters carbon from the ocean–atmosphere system^23^. This reaction proceeds more rapidly with higher global temperatures and associated intensification of the hydrologic cycle. The result is a stabilizing feedback that is widely viewed to give rise to the persistence of clement climates on Earth^14,20^. Within this canonical framework, it is expected that rapid CO_2_ release will be met by accelerated rates of silicate weathering and thus enhanced carbon removal, stabilizing and restoring temperatures on 10^5^ yr timescale^14,15,24^ (e.g., Paleocene–Eocene event^3,25^). In contrast, subsequent to volcanic carbon release and rapid temperature rise at the end-Permian^26^, temperatures^2^ and CO_2_ levels^27^ remained elevated for over five million years (Myr)—an anomalously protracted interval. Here, Earth’s climate system “failed” to recover over the expected timescale^3^ (Fig. 1B; over an order of magnitude more protracted than well-constrained Cenozoic climate perturbations^3,25^). Instead, the Early Triassic appears to be characterized by a semi-stable hyperthermal state^2,3^. Biotic recovery was also stifled for this extended >5 Myr interval in the wake of the mass extinction, coincident with the interval of sustained warmth^2,28,29^. Although elevated temperatures and widespread anoxia were likely key factors in prolonging the end-Permian biotic crisis^13^, here we make the case that biotic regulation of climate may have also played a critical role in sustaining elevated greenhouse conditions.

**Fig. 2:**
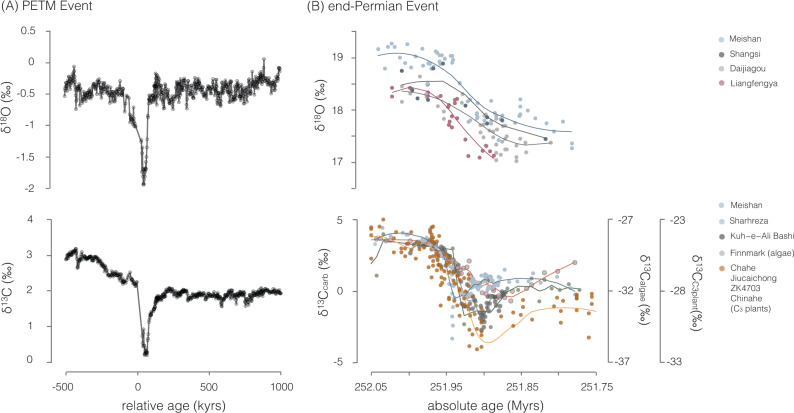
Carbon and oxygen isotope records. Carbon-isotope (δ^13^C) and oxygen isotope (δ^18^O) records across the (**A**) Paleocene–Eocene Thermal Maximum (PETM; ODP Site 1262) and (**B**) end-Permian. Latest Permian and Early Triassic δ^13^C archives include carbonate^32,88,89^, algae^8^, and plant matter^4^. Negative carbon-isotope excursions are traditionally interpreted to mark the release of isotopically light carbon, while oxygen isotopes are used as a paleo-temperature proxy and are measured in conodont elements for the end-Permian event and in biogenic carbonate (planktonic foraminifera and coccoliths) for the PETM^2,3,32,90^. The PETM exhibits tight stratigraphic covariation between the δ^13^C and δ^18^O isotope excursions, in contrast to end-Permian records.

Geochemical records indicate that solid-Earth degassing was unlikely the sole factor shaping global climate during this event. Foremost, the prolonged warm interval extends well beyond estimates for the timing of active carbon release (estimated <0.4 Myrs) from the Siberian Traps and adjacent coals^7,8,26,30,31^. Further, a decoupling between reconstructed temperatures and carbon outgassing rates during the initial temperature rise (based on oxygen and carbon isotopes respectively^32,33^ from the Meishan section (South China)) was recently highlighted as potentially deviating from other well-characterized hyperthermal events such as the Paleocene–Eocene Thermal Maximum (PETM) even when accounting for the longer and therefore more severe climatic perturbation at the end-Permian (Fig. 2)^3^. Additional multi-million-year climate forcing(s) and/or a fundamentally altered carbon cycle are therefore required to explain these observations. Here, we demonstrate that a shift in the extent of authigenic clay formation can provide an explanation for the decoupling of these geochemical records.

In this study, we investigate the role that the decline of silica-secreting organisms may have played in driving anomalous climate behavior in the wake of the end-Permian mass extinction, through mineralogical analysis of marine shales and carbon–silica modeling.

## Results and discussion

### Carbon–silica cycle modeling

**Fig. 3:**
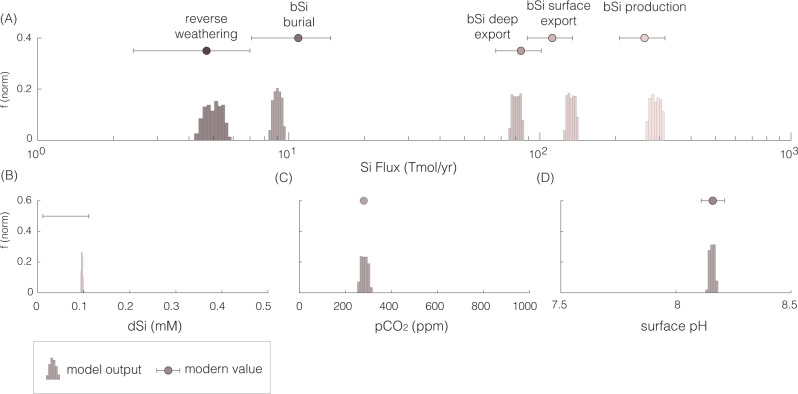
Carbon–silica cycle model calibration. The modern-day values of (**A**) marine silica fluxes including biogenic silica (bSi) production, surface to deep export, deep to sediment export, sediment burial, and reverse weathering, **B** surface-dissolved silica levels, **C** preindustrial atmospheric CO_2_ levels and (**D**) surface-seawater pH are reproduced by our model. Parameters utilized include *n*_si_ = 0.2, *n*_carb_ = 0.1, ${{p}{{{{{{\rm{CO}}}}}}}}_{2}^{c}$ = 140, *r*_si_ = 3, *r*_H_ = 1, and Alk:Si = 0.3. Vertical axis indicates frequency (normalized) of the Monte Carlo results.

To quantitatively investigate climate dynamics (and associated feedbacks) during the latest Permian through the Early Triassic, we utilized a coupled carbon and silica (Si) global biogeochemical mass balance model. Foremost, we calibrate the model to reproduce the most recent constraints of the modern global marine silica cycle fluxes^34^ (including internal recycling of silica; Fig. 3). We adopted a stochastic approach for error propagation (Monte Carlo sampling) of standard parameters including silicate weathering, reverse weathering, extinction duration, climate sensitivity (temperature change with a doubling of *p*CO_2_), and a volcanic plus associated sediment outgassing range of CO_2_ input that encompasses the full range proposed from previous studies^7,30,31^ (Supplementary Tables 2 and 3).

In Simulation 1, we quantified the effects of volcanic CO_2_ release on Earth’s climate. The results indicate that both peak temperatures and the long-term Early to Middle Triassic plateau are not reproducible (Fig. 1A, B) with previously proposed volcanic CO_2_ release rates and any standard representation of the silicate weathering feedback (i.e., the typical range of silicate and carbonate weathering exponents adopted in global carbon cycle models; see “Methods”). This quantitatively supports the idea that the observed sustained warmth following the end-Permian crisis indicates a silicate weathering feedback that may have been ineffective at drawing down *p*CO_2_ and lowering temperatures^3^.

It has been postulated that the volcanic carbon injection may have been large enough to saturate the capacity of terrestrial silicate weathering to act as a stabilizing feedback on *p*CO_2_, providing a mechanism to explain the sustained Triassic warmth^3^. Specifically, substrate limitation would have inhibited carbon sequestration via silicate weathering. However, previous attempts to quantify total available alkalinity from primary silicates (compared to outgassing estimates) likely represent low end-member values, given that both groundwater^35^ and marine weathering^36^ sources of alkalinity were not considered. More importantly, preserved Lower Triassic palaeosols have not been congruently weathered (i.e., the preserved soils still bear soluble cations and could potentially have undergone more extensive chemical weathering)^37,38^. In this light, the latest Permian and Early Triassic climate behavior necessitated an Earth system response other than (or in conjunction with) substrate limitation.

Here, we demonstrate (in a second simulation; Simulation 2) that the demise of the biological silica factory and a corresponding increase in the degree of carbon recycling tied to authigenic clay formation^39^ can account for the anomalous climate behavior observed during the end-Permian crisis. Authigenic formation of clays in the marine realm converts carbonate alkalinity (HCO_3_^−^) back into carbon dioxide^19,40,41^. This process, commonly referred to as reverse weathering, acts to elevate atmospheric *p*CO_2_ levels by retaining (recycling) carbon within the ocean–atmosphere system^19,40,41^. Consequently, this will increase the residence time of carbon within the ocean–atmosphere reservoir. The result is a CO_2_ source that bears the isotopic signature of contemporaneous seawater with essentially no unique carbon isotopic footprint—satisfying a key requirement for resolving the decoupling between temperatures and solid-Earth degassing (Fig. 2). A secondary effect of an increase in CO_2_ release from reverse weathering is an increase in terrestrial weathering intensity (particularly of carbonates) that acts to drive the δ^13^C of the ocean and atmosphere towards more positive values. Combined, both these effects would have allowed for the persistence of a relatively large atmospheric CO_2_ reservoir that does not bear a low carbon-isotope signature.

As in previous studies^19,42^, reverse weathering can be simply represented as the ratio of Si being sequestered as an authigenic clay to the total amount of Si removed (*f*_rw_). Here, we distinguish between the ratio observed in sediments from two sections in Japan that span the Permian/Triassic boundary and that of our model outputs. The ratio expressed in sedimentary records (*f*_rw_s_) reflects the mass-weighted authigenic and detrital components, each with its own ratio, *f*_rw_s-a_ and *f*_rw_s-d_ respectively. Modeled outputs (e.g., C–Si cycle modeling results of Fig. 1) provide an authigenic estimate (defined as *f*_rw_m-a_) required to explain the temperature trend across the end-Permian mass extinction and the sustained early-Triassic warm interval. For direct and meaningful comparisons between the authigenic components of the sedimentary and modeled outputs (i.e., *f*_rw_s-a_ and *f*_rw_m-a_), we adopt a simple mixing model (see “Methods”) that estimates *f*_rw_s-a_ from the ratio expressed in sediments (*f*_rw_s_).

### Sedimentological evidence for a modal shift in silica export

**Fig. 4:**
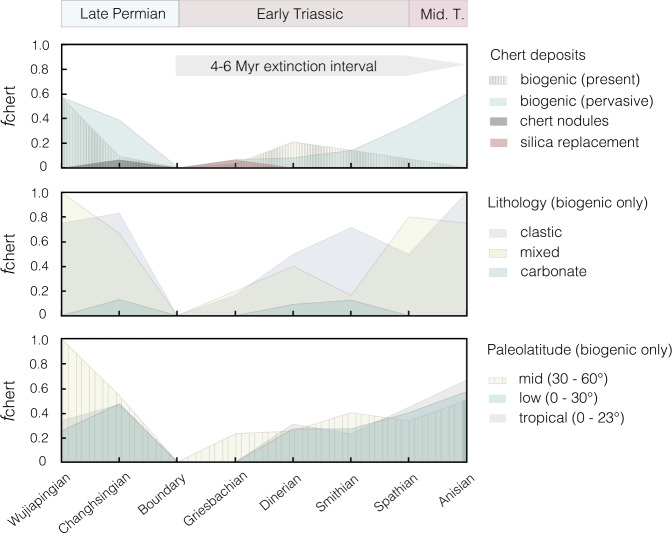
The fraction of marine sedimentary sections bearing chert (*f*_chert_), spanning the Late Permian to Middle Triassic. The global compilation includes a total of 36 geographically disparate sites spanning a broad range of paleolatitudes (0–72°) (see Supplementary Fig. 1 and Supplementary Table 1). **A** Chert deposit types include biogenic (e.g., sponge spicules or radiolarian noted), nodules, and silica replacement. Biogenic chert deposits according to (**B**) lithology and (**C**) paleolatitudes.

There is abundant evidence from the geologic record for a dramatic perturbation to the global silica cycle at the end-Permian. Perhaps most prominently, a pronounced collapse in both the diversity and abundance of siliceous organisms at the end-Permian crisis—commonly referred to as the Early Triassic “chert gap”—has been described in sections worldwide^1,39,43–45^. Here, we present a global compilation (36 localities) of sedimentary chert (biogenic silica, nodules, and silica replacement) occurrences across the Late Permian to Middle Triassic interval that highlights this regime shift (Fig. 4 and Supplementary Fig. 1). A decrease in the presence and/or preservation of chert is prominent within sections of all different lithological types (siliciclastic, carbonate, and mixed lithologies) and paleolatitude bands. Notably, this feature is in direct contrast with records of Jurassic and Cenozoic warming events that have instead been shown to trigger pulses of chert deposition^25,46,47^. In the traditional representation of the silicate weathering feedback, every mole of carbon dioxide consumed corresponds to roughly one equivalent mole of silica delivered to the oceans^14,23^. On this basis, increased CO_2_ degassing is expected to dramatically elevate chert deposition, as opposed to the observed Early Triassic lull—a feature that currently lacks explanation.

As a result of both a dramatic reduction in biogenic chert deposition and enhanced silicate weathering, our modeling results demonstrate that the global marine silica inventory would have begun to rapidly accumulate within ~5000 years (Fig. 1 and Supplementary Figs. 4 and 8). There are only two burial pathways for silicon, silica (biogenic or abiogenic SiO_2_) and marine clay formation. An increase in dissolved silica, tied to the decimation of silica biomineralizers, will enhance abiotic depositional pathways by increasing the saturation states of these phases until a new steady state is achieved. This scenario resembles a Precambrian-like end-member regime during which siliceous organisms had not yet evolved and a greater fraction of total silica output was sequestered through reverse weathering^19,42,48^. Increased primary productivity and widespread anoxia—well-established features of the end-Permian to Early Triassic biotic crisis—could have played a role in promoting clay formation. An increase in the proportion of anaerobic (as opposed to aerobic) organic carbon degradation (e.g., through sulfate reduction) within marine and sedimentary porewater produces alkalinity that would have further promoted clay formation during this time^49^.

**Fig. 5:**
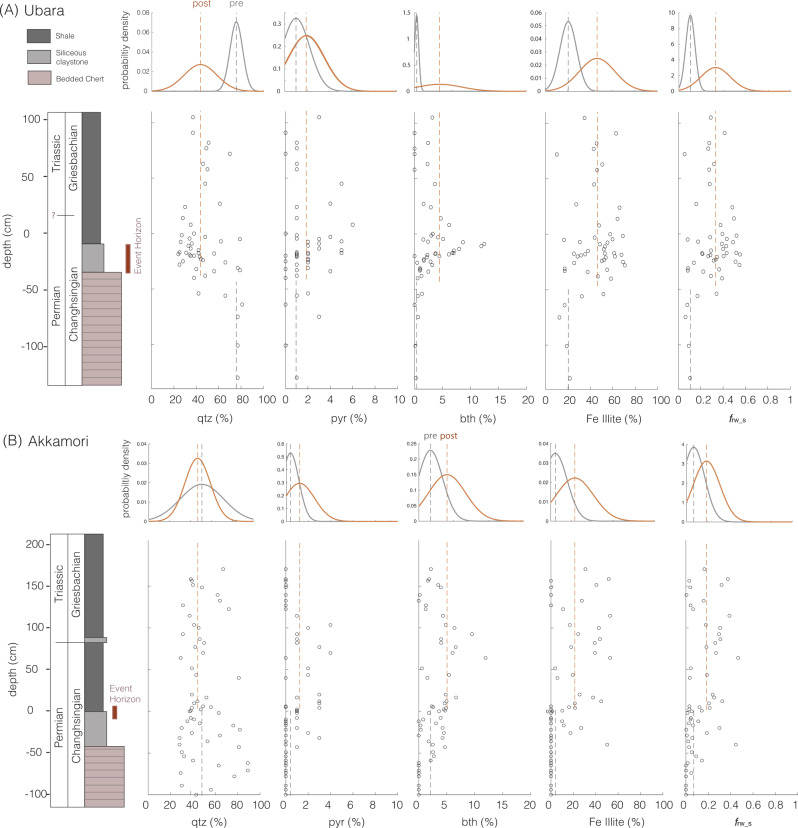
Mineralogical data across the extinction event. Mineralogical data from (**A**) Ubara and (**B**) Akkamori. Quartz (qtz); pyrite (pyr); berthierine (bth); Fe-illite; and *f*_rw_s_.

**Fig. 6:**
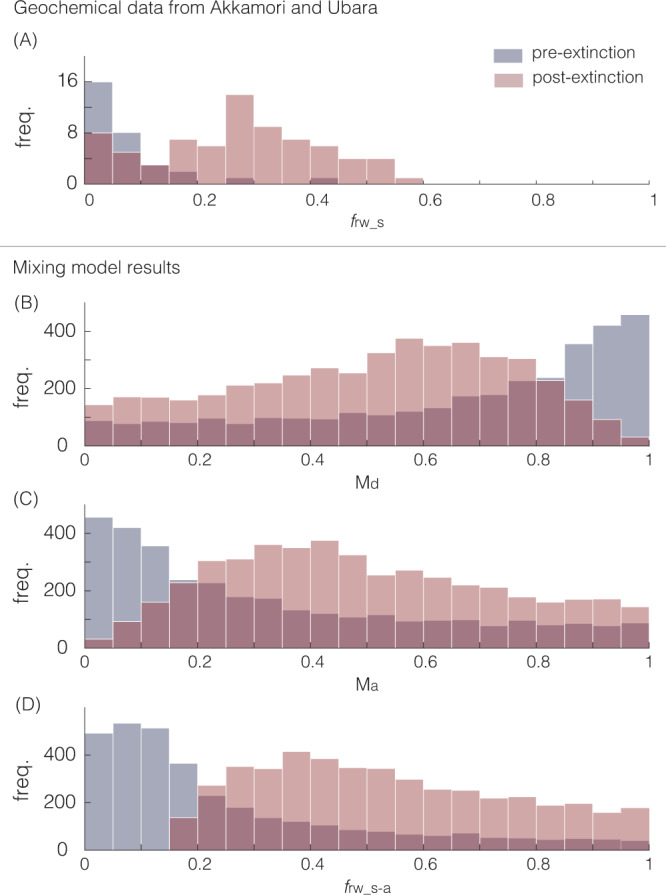
*f*_rw_s_ and sediment mixing model results. **A** Combined *f*_rw_s_ from Akkamori and Ubara; **B**–**D** filtered mixing model results consistent with the *f*_rw_s_ shift across the event; **B** detrital mass fraction, M_d_; **C** authigenic mass fraction, M_a_; **D** *f*_rw_s-a_.

Critically, these results appear consistent with sedimentological evidence for a spike in marine dissolved silica levels across the extinction boundary. Foremost, recent work has described extensive silicification of marine fossil assemblages observed from the Griesbachian^50,51^. Here, we provide additional mineralogical support from two siliciclastic deep-sea sections Akkamori and Ubara, Japan (stratigraphy and age constraints are detailed in refs. ^39,52^) both highlighting an increase in the abundance of cation-bearing clay minerals (namely berthierine, Fe-smectite, and Fe-illite) coincident with the loss of bedded chert across the extinction boundary (Fig. 5). The mineralogical data also reveal a shift in estimated *f*_rw_s_ from 0–0.15 pre-extinction to 0.15–0.55 post extinction (Fig. 6A), consistent with an increase in the rate of reverse weathering across the event. As a ratio, *f*_rw_ is not influenced by variations (if any) in sedimentation rate (i.e., the ratio is not subject to dilution effects). Using a simple mixing model (see “Methods”), we estimate that an increase in *f*_rw_s-a_ across the extinction horizon from 0–0.2 to 0.2–0.65 (with a peak at ~0.4; Fig. 6D) is required to explain the observed *f*_rw_s_ expressed in the sediment record (Fig. 6A).

### Carbon recycling

To quantitatively assess if anomalous Early Triassic climate trends can be reconciled by considering potential changes in carbon recycling driven by a decline in biogenic silica deposition, we build directly from Simulation 1 where the authigenic clay flux is invariant (Fig. 1A, B and Supplementary Figs. 2–4). In Simulation 2, authigenic clay formation is allowed to vary dynamically over the course of the extinction event depending on clay solubility in seawater, as regulated by dissolved silica and pH levels (see “Methods”). Based on first principles (thermodynamics and kinetic drivers), higher silica and pH levels act to promote clay authigenesis e.g. ref. ^53^. A wide range of parametrization was explored as part of the Monte Carlo simulation. In particular, an alkalinity to silica consumption ratio of 0.17–6 (corresponding to a 1st to 6th order dependence of clay authigenesis on [H^+^] and [Si]) is explored to reflect the full range of globally integrated clay mineral assemblages possible in marine sediments (i.e., not based on a single mineral; see “Methods”). Given that authigenic clays are often found in association with biogenic silica dissolution and alteration in modern oceans^54–59^, we also consider the effect of biogenic silica alteration. Specifically, a biogenic term regulated by the extent of biogenic silica sediment export is included as part of the proportionality term for reverse weathering, such that any variations to biogenic silica export will have a direct influence on reverse weathering (see “Methods”).

**Fig. 7:**
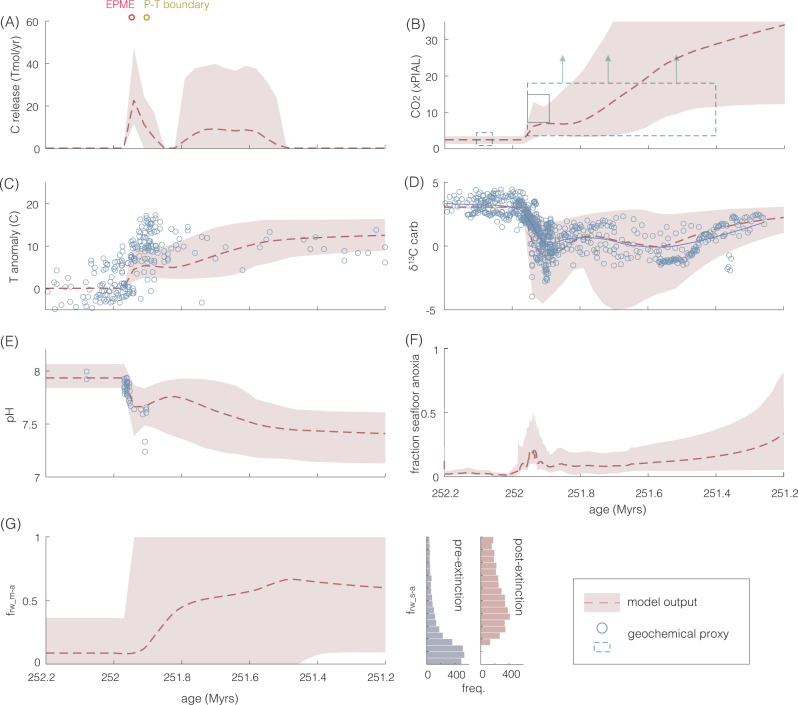
Successful modeling results from Simulation 2 reproducing geochemical proxy data at the end-Permian mass extinction (EPME). Red dashed line indicates the average model output, and 95% of the data falls within the pink envelope. Input parameters follow that of Fig. 1. **A** Carbon released from solid Earth (volcanic) and sedimentary metamorphic degassing fluxes (Tmol/yr); **B** atmospheric *p*CO_2_ (×PIAL) levels, dashed gray boxes indicate proxy estimates from stomata^60,61^, paleosol carbonates^62,63^, phytane^64^ and δ^13^C of plant matter^4^; the upper estimate of the dashed boxes indicate the minimum values (as indicated by the up arrows) given that both stomatal index and the Δδ^13^C plant proxy saturate at ~800 and 2500 ppm, respectively^91,92^ (i.e., proxies are not sensitive to higher *p*CO_2_ beyond this level); the solid gray box indicates estimates from boron isotope data^11^; **C** temperature anomaly, sea surface temperature reconstructions (blue circles)^2,32,33^ with age constraints based on^9,87^; **D** δ^13^C_carb_ (ref. ^4^), model reconstruction represent the δ^13^C of surface DIC offset by +0.5 ‰; **E** marine pH (ref. ^11^); **F** fraction seafloor anoxia derived from^93^; **G** *f*_rw_m-a_ (left) and *f*_rw_s-a_ (right) as presented in Fig. 6.

This addition of a dynamic reverse weathering flux allows for a more realistic representation of global carbon–silica cycle feedbacks. Specifically, the reverse weathering feedback is also considered in addition to the silicate weathering feedback. The raw model results (Supplementary Figs. 5 and 6) are then filtered against the observed temperature trends across the end-Permian event. Upper and lower bounds on the temperature filter were set at ±4 °C and ±0.4 Myr from the mean. Successful runs that reproduced the temperature record were obtained from Simulation 2 (Fig. 1C–L and Supplementary Figs. 7 and 8), in direct contrast to results from Simulation 1 where there were no successful model runs. While identical volcanic forcing (carbon release) was prescribed for both simulations (1 and 2), very distinct climatic and environmental (temperature, pH) outcomes were generated (Fig. 1 and Supplementary Fig. 2). Notably, successful runs from Simulation 2 also reproduce the reconstructed pH, *p*CO_2,_ and δ^13^C trends observed across the end-Permian^4,11,60–64^ (Fig. 7).

Non-steady-state behavior characterizes the immediate aftermath of the extinction (Fig. 1C–L). Across the boundary, increasing dissolved silica levels act to promote clay authigenesis while declining pH acts to inhibit it. Despite these opposing effects, the results indicate a steady rise in clay authigenesis following the extinction. Inorganic silica deposition follows an identical trajectory with a slight temporal lag. Subsequently, the Earth system reaches a new equilibrium (steady state) spanning the anomalous 4–6 Myr Early Triassic warm period, through both the silicate weathering^15^ and reverse weathering^19^ stabilizing feedbacks. Here, clay authigenesis and inorganic silica burial reach a plateau. The successful results from Simulation 2 indicate that a shift to more elevated *f*_rw_s-a_ values between 0.17 and 0.6 relative to pre-extinction values of <0.18 (equivalent to a rise in clay silica uptake by >9% of total silica export—16th percentile) is sufficient to sustain the prolonged greenhouse climate (Fig. 1K).

This result is consistent with the *f*_rw_s-a_ range derived from the mixing model based on the *f*_rw_s_ (as determined from XRD data) expressed in our examined sedimentary sections across this interval (Fig. 6). This shift in the balance of silicate weathering and reverse weathering corresponds to an increase in the residence time of carbon by 2.2–4.4-fold, highlighting the recycling effect of clay formation and strengthening the assertion that clay authigenesis helped sustain the post-extinction warm interval (Fig. 1L). These results directly contrast with the minimal change in carbon residence time observed in Simulation 1 (Supplementary Fig. 2J). Results from Simulation 2 also highlight the development of carbonate supersaturation in seawater (Supplementary Fig. 7J), consistent with the appearance of giant ooids and authigenic carbonate textures that indicate elevated rates of carbonate precipitation^65–68^.

Combined, geochemical records and modeling results provide support for enhanced carbon retention via marine reverse weathering as a viable solution for anomalous Early Triassic climate trends. Notably, this model framework provides, for the first time, a self-consistent mechanistic link between the lethally hot temperatures, delayed climate recovery, sustained low pH conditions, widespread loss of silicifying organisms, carbonate supersaturation, and anomalous silica cycle behavior at the end-Permian without necessitating additional solid-Earth sources of greenhouse gases. This indicates that the ecological success or demise of siliceous organisms across a climate perturbation can potentially play a major role in controlling both the severity and duration of an environmental crisis.

The end-Permian mass extinction is, to our knowledge, the only Phanerozoic carbon injection event that resulted in the widespread disappearance of siliceous organisms and cessation of biotic silica deposition. As the only historical event with carbon release rates that may be similar to the modern^6^—despite the significant difference in the durations of these events—the end-Permian provides an impetus to better quantify modern reverse weathering rates and the potential for sluggish CO_2_ drawdown over the upcoming millennia—in view of the anticipated changes in marine sediment fluxes, deoxygenation, primary productivity, and potentially Si levels. Despite extensive focus on how carbonate secreting organisms are impacted by and in turn regulate the global carbon system during climate perturbations, significantly less attention has been paid to siliceous organisms. Notably, recent work demonstrated significantly reduced silica uptake in diatoms (today’s dominant silica secreting organism) at *p*CO_2_ levels predicted for the end of the century^69^. This promotes the need to better quantify the synergistic effects of environmental change (warming, deoxygenation, and acidification) on siliceous organisms and vice versa, so as to develop a more complete understanding of how marine ecosystems respond to and shape climatic events.

## Methods

### “Chert gap” compilation

The data compilation of Late Permian to Middle Triassic marine sections in Fig. 4 is provided in Supplementary Table 1. Listed localities are generally characterized by a stratigraphic section. The presence of chert was noted based on descriptions of silica, chert, radiolarians, or sponge spicules in the text or stratigraphic columns. To determine whether the “chert gap” appears to be a feature of preservation bias, we compiled records from carbonate and siliciclastic sections. To determine whether environmental changes related to latitudinal positioning were responsible, we also compared the presence of chert across paleogeographic regions. The reported lithology is based on the predominant lithology reported for each stratigraphic section. Paleolatitude constraints were obtained using reported modern coordinates and reconstructed using the PALEOMAP PaleoAtlas for GPlates (EarthBytes) and GPlates v. 2.1.0 (ref. ^70^). Supplementary Fig. 1 indicates the total number of sites per time bin. The abundance of biogenic chert appears to recover most rapidly in the high latitude (60–90°) interval. However, it should be noted that this interval is represented by only a single site (New Zealand). Sites at tropical, low, and mid-paleolatitudes indicate a relatively delayed recovery (by ~Spathian–Anisian) relative to background values.

### Extinction phases

The extinction of calcareous and siliceous taxa was implemented in three distinct phases as part of the Monte Carlo simulation. (1) The initiation phase (10^5^ yrs, i.e., time between the start of the extinction and the full extinction). (2) The full-extinction interval (2–7 Myrs), with suppressed biogenic calcite and chert deposition. (3) The recovery phase (1–6 Myrs; time between the full extinction and recovery). The initiation and recovery phases are parameterized linearly (between background and full-extinction deposition).

### Global carbon–silica cycle model

For this work, we adopted a modified global carbon–silica cycle model^71^. The overall structure of this model follows widely adopted carbon^72^ and silica^25,73,74^ cycle models. This fully coupled global carbon–silica cycle keeps track of all the relevant geochemical tracers for modeling long-term carbon and silica cycling. Specifically, this includes carbon species as atmospheric *p*CO_2_ and DIC in seawater, dissolved silicon (DSi), and seawater total alkalinity (TA). The ocean reservoir is divided into two boxes: the surface (top 100 m) and deep (>100 m). The former is coupled to the atmosphere reservoir of CO_2_. All model configurations were first spun up to stable equilibrium until tracers in all reservoirs achieved steady state, before the initiation of any perturbations. For a more comprehensive investigation of global carbon–silica cycling and its influence on climate at the end-Permian, we have modified the model to include; (1) a Monte Carlo sampling approach of input parameters for error propagation; (2) silica fluxes from groundwater, marine weathering and terrestrial SiO_2_ weathering; (3) internal recycling of biogenic silica in the marine realm (production, dissolution, export and burial) to achieve a fully comprehensive representation of marine silica cycling; (4) organic carbon burial and terrestrial organic carbon weathering; (5) ^13^C as a tracer (the air sea gas exchange term for ^13^C is set up as described by Zeebe, 2012 for LOSCAR^72^); and (6) reverse silicate weathering (ocean–atmosphere carbon recycling CO_2_ associated with authigenic clay formation), as detailed below.

Constant silica fluxes into the marine realm include dust (*F*_dust_), hydrothermal (*F*_hyd_), marine sediment silicate weathering (*F*_mssw_), and groundwater (*F*_gw_) sources (see Supplementary Table 2).

Dynamic silicate fluxes include silicate, carbonate, and quartz weathering. Silicate and carbonate weathering are parameterized according to the canonical expression:

1 $${F}_{{{{{{\rm{sillw}}}}}}}={F}_{{{{{{\rm{silw}}}}}}}^{0}{{\cdot }}{\left(\frac{{p{{{{{\rm{CO}}}}}}}_{2}}{{p{{{{{\rm{CO}}}}}}}_{2}^{c}}\right)}^{{n}_{{si}}}$$

2 $${F}_{{{{{{\rm{carbw}}}}}}}={F}_{{{{{{\rm{carbw}}}}}}}^{0}{{\cdot }}{\left(\frac{{p{{{{{\rm{CO}}}}}}}_{2}}{{p{{{{{\rm{CO}}}}}}}_{2}^{c}}\right)}^{{n}_{{carb}}}$$

as adopted in previous carbon cycle models (e.g., refs. ^14,15,72^), where ${F}_{{{{{{\rm{silw}}}}}}}^{0}$ and ${F}_{{{{{{\rm{carbw}}}}}}}^{0}$ represent the initial silicate and carbonate weathering fluxes, respectively. ${F}_{{{{{{\rm{silw}}}}}}}^{0}$ is set to equal the background volcanic CO_2_ degassing flux (*F*_vc_). *p*CO_2_ and ${p{{{{{\rm{CO}}}}}}}_{2}^{c}$ are the real-time and constant values respectively. The long-term steady-state *p*CO_2_ value is in part determined by the ${p{{{{{\rm{CO}}}}}}}_{2}^{c}$ value. Here, we explore a range of ${p{{{{{\rm{CO}}}}}}}_{2}^{c}$ values as part of the Monte Carlo simulation. Steady state is achieved when the *p*CO_2_ value equals ${p{{{{{\rm{CO}}}}}}}_{2}^{c}$ if there is no alkalinity consumption (carbon recycling) tied to authigenic clay formation. Any nonzero amount of alkalinity consumption tied to reverse weathering will act to elevate atmospheric *p*CO_2_ and silicate weathering. In this system, steady state is achieved at *p*CO_2_ > ${p{{{{{\rm{CO}}}}}}}_{2}^{c}$ as a higher flux of silicate weathering is required to balance volcanic carbon input. The weathering exponents *n*_si_ and *n*_carb_ regulate the strength of the weathering feedback^72^. Here, we explore a broad range of previously proposed weathering exponents^75^ (*n*_si_ = 0.2–0.5; *n*_carb_ = 0.1–0.3), as highlighted in Supplementary Table 3. Steady-state pre-extinction (background) atmospheric CO_2_ levels of between 300 and 1000 ppm are selected during the model spin-up (i.e., any individual spin-up with a final steady-state value that fell outside this range was repeated until an acceptable steady-state value within the prescribed range was met).

The terrestrial weathering of SiO_2_ (*F*_SiO2w_; e.g., quartz, sandstone, chert) is set at 19% of the silicate weathering flux (*F*_sillw_). Of the total 8.1 Tmol yr^−1^ dissolved silica transported to the marine realm through rivers today^34^, modeling results estimate that ~6.8 Tmol yr^−1^ is driven by silicate weathering. Here, 5 Tmol yr^−1^ matches the volcanic degassing and a further 1.8 Tmol yr^−1^ increase is associated with the recycling of carbon tied to reverse weathering (with an Alk:Si consumption ratio of 0.5). The remaining flux is derived from the weathering of quartz, at 1.3 Tmol yr^−1^ (which equates to 16% of the total riverine flux).

Silica output fluxes from the marine realm include inorganic silica, biogenic silica and authigenic clay formation. Biogenic silica cycling here includes production, export, dissolution, and burial. Biogenic silica production (*F*_bSip_) is parameterized according to a Michaelis–Menten kinetics function:

3 $${F}_{{{{{{\rm{bSip}}}}}}}={k}_{{{{{{\rm{bSi}}}}}}}{{\cdot }}\frac{{V}_{{{\max }}}{{\cdot }}{\left[{Si}\right]}_{{ssw}}{{\cdot }}1000}{{{K}_{m}+\left[{Si}\right]}_{{ssw}}{{\cdot }}1000}$$

where *V*_max_ = 1.74 and *K*_m_ = 74.48 (ref. ^76^) and *k*_bSi_ is the proportionality constant with values of 27 × 10^13^ to 32 × 10^13^, reproducing modern-day dissolved silica levels and C–Si fluxes (Fig. 3). Of the three dominant groups of bio-silicifying organisms today, sponges, radiolarians, and diatoms, only the first two had evolved by the Permian. Notably, both sponge and radiolaria silica production have been proposed to scale with ambient silica availability^76,77^. A lower range of *k*_bSi_ values of 0.1 × 10^11^ to 7 × 10^13^ are adopted for the end-Permian simulations, based on the long-standing view that biogenic silica uptake may not have been as efficient as the modern system^78,79^, allowing for a broader range of elevated background steady-state dissolved silica levels to be explored.

Biogenic silica dissolution (*F*_bSid_) in each reservoir is parameterized according to:

4 $${F}_{{{{{{\rm{bSid}}}}}}}={F}_{{{{{{\rm{bSiin}}}}}}}{{\cdot }}{\left(1-\frac{{\left[{Si}\right]}_{{{{{{\rm{sw}}}}}}}}{\varsigma }\right)}^{\rho }$$

where *F*_bSiin_ is the input flux of biogenic silica into the reservoir (e.g., surface production, or export from surface-deep and deep-sediment), and *ς* = 0.9 mM is the solubility constant for biogenic silica (opal-A)^80,81^. A *ρ* value of 5.5 is adopted for surface and deep marine reservoirs, and 0.4 is assigned for sediment dissolution flux back into the deep ocean reservoir. We find that these values are capable of reproducing the most recent constraints of the modern-day global marine silica cycle fluxes^34^ (Fig. 3).

Authigenic clay export is parameterized according to:

5a $${F}_{{{{{{\rm{rw}}}}}}}=\left(\beta +\alpha \right){{\cdot }}({\Omega }_{{{{{{\rm{rw}}}}}}}-o)$$

where the proportionality constant term $\alpha ={F}_{{{{{{\rm{vc}}}}}}}+{F}_{{{{{{\rm{hyd}}}}}}}+{F}_{{{{{{\rm{dust}}}}}}}+{F}_{{{{{{\rm{mssw}}}}}}}+{F}_{{{{{{\rm{gw}}}}}}}$, and the biogenic term $\beta ={F}_{{{{{{\rm{bSi}}}}}}\_{{{{{\rm{sed}}}}}}}.\frac{{F}_{{rw}}^{{mod}}}{{F}_{{bSi\_sed}}^{{mod}}}$ is the product of the modeled biogenic silica flux to marine sediments (*F*_bSi_sed_) and the ratio of the modern reverse weathering flux (${F}_{{rw}}^{{mod}}$ = 4.7 ± 2.3 Tmol yr^−1^) to the modern biogenic silica export (planktonic + benthic) flux to marine sediment (${F}_{{bSi\_sed}}^{{mod}}$ = 90.2 ± 19.1 Tmol yr^−1^). Here, we explore the full range of possible $\frac{{F}_{{rw}}^{{mod}}}{{F}_{{bSi\_sed}}^{{mod}}}$ from 0.022 to 0.099 as part of the Monte Carlo. The biological enhancement term is incorporated in view of work highlighting the association of authigenic clays with biogenic opal alteration/dissolution in modern marine sediment^54–59,82^. A value of 1 is adopted for the constant term *o* for the modern-day calibration, while a broader range of values of 1–7 are explored as part of the Monte Carlo for the end-Permian simulations. The saturation index Ω_rw_ is estimated as follows:

5b $${\Omega }_{{{{{{\rm{rw}}}}}}}=\frac{{{{{{\rm{IAP}}}}}}}{{{{{{{\rm{K}}}}}}}_{{sp}}}$$

5c $${{{{{\rm{IAP}}}}}}=\frac{{[{{{{{\rm{X}}}}}}]}_{{{{{{\rm{ssw}}}}}}}^{{r}_{x}}{{\cdot }}{[{{{{{\rm{Si}}}}}}]}_{{sw}}^{{r}_{{si}}}}{{[{H}^{+}]}_{{sw}}^{{r}_{H}}}$$

5d $${{{{{{\rm{K}}}}}}}_{{sp}}=\frac{{[{{{{{\rm{X}}}}}}]}_{{{{{{\rm{o}}}}}}}^{{r}_{x}}.{[{{{{{\rm{Si}}}}}}]}_{{{{{{\rm{o}}}}}}}^{{r}_{{si}}}}{{[{{{{{{\rm{H}}}}}}}^{+}]}_{{{{{{\rm{o}}}}}}}^{{r}_{H}}}$$

here, [Si]_sw_ and [H^*+*^]_sw_ represent the seawater silica and H^+^ concentrations, and [X] represents a generic cation (mono/di/tri-valent) compatible with authigenic clay minerals (e.g., Mg^2+^, Fe^2+^, Fe^3+^, K^+^). For simplicity, a value of 1 is applied for both [X] and its exponent *r*_x_ in this study. *r*_si_ = 1 and *r*_H_ = 1 to 2 are adopted for the pre-extinction interval. In order to reflect the full range of possible globally integrated clay minerals assemblages (i.e., not based on a single mineral) expressed in marine sediments for the extinction interval, we explore a broad range of values for the exponents *r*_si_ = 1 to 6 and *r*_H_ = 1 to 6 as part of the Monte Carlo sampling (Supplementary Table 3). K_*sp*_ constants of [H^+^]_o_ = 1.9 × 10^−5^ mM and [Si]_o_ = 0.123 mM are selected to reproduce the modern reverse weathering silica uptake flux (Fig. 3), with alkalinity to silica ratio (Alk:Si) of ~0.3 (consistent with results from modern experimental work^83^).

The alkalinity to silica consumption ratio (Alk:Si) of the globally integrated authigenic clay assemblage links the global carbon and silica cycles. To reproduce the pre-extinction (Permian) carbon, silica, and CO_2_ levels, Alk:Si values of 1–2 are adopted (consistent with the occurrence of glauconite noted in pre-extinction interval of the section examined here). The sedimentary records indicate that Alk:Si could have evolved across the event, and so a broader (full) range of possible Alk:Si values, from 0.17 to 6, as exhibited by common clay minerals in the geologic record is explored for the duration of the extinction as part of the Monte Carlo. Notably, the mineral berthierine that is common in the post-extinction interval of our sections presented here has an Alk:Si value of ~4.

The flux of inorganic silica is parameterized according to:

6 $${F}_{{{{{{\rm{iSi}}}}}}}=(\beta +\alpha ).{\left(\frac{{\left[{Si}\right]}_{{{{{{\rm{ssw}}}}}}}}{\partial }-1\right)}^{1.1}$$

where *∂* is a constant below which there is no abiotic silica formation (if [*Si*]_ssw_ < ∂, *F*_iSi_ = 0). A range of ∂ between 0.6 and 0.9 mol m^−3^ was explored in the model simulations^80,84^ (Supplementary Table 3).

### Coupled C–Si cycle simulations

As described in the main text, we performed two model simulations. *Simulation 1: degassing + fixed carbon recycling:* In Simulation 1, volcanic (solid Earth) release of carbon and biotic extinction are the only prescribed forcings. Ranges of input parameters are listed in Supplementary Tables 2 and 3. Here, the marine silica cycle does not have any dynamic (transient) influence on the carbon cycle, as authigenic clay formation is kept at a constant value through mass extinction. An increase in *p*CO_2_ drives an increase in silicate weathering and thus dissolved silica concentrations, that in turn increases inorganic silica formation (Supplementary Figs. 2–4). *Simulation 2: degassing + dynamic carbon recycling:* All input parameters are kept the same as in Simulation 1. Simulation 2 builds from Simulation 1 in that authigenic clay formation is allowed to vary over time (dynamic carbon recycling). Unfiltered model results are presented in Supplementary Figs. 5 and 6 and filtered results are presented in Supplementary Figs. 7 and 8.

The residence time of carbon within the ocean–atmosphere system (reservoir size/export fluxes) is regulated by variations in inputs (volcanic degassing), outputs (carbonate export), and recycling (reverse weathering) within the model. The differences between the results presented for Simulation 1 and 2 provide us with useful insight into how changes in the flux of authigenic clay formation influences the global carbon cycle. The rapid rise at the onset in both simulations is driven by the combined effect of both the carbon injection that acts to increase the total carbon in the ocean–atmosphere system, and a crash in biogenic carbonate export. Subsequently, a new steady state is reached at an elevated carbon residence time for Simulation 2, reflecting the enhanced carbon recycling that sustained the warm interval, while in Simulation 1 background levels are quickly reestablished.

We find that a full-extinction duration of as short as 2 Myr yields successful results from Simulation 2 (Supplementary Fig. 9 displays the distributions of raw and filtered results). Durations of >3.8 Myrs have similar frequency of success, and durations of <3 Myrs have a relatively lower success frequency.

### XRD analysis

X-ray diffraction (XRD) analysis was carried out using a PANalytical Empyrean diffractometer, employing a Co Kα source and PIXcel-1D detector at the University of Oxford. Each sample was prepared as a randomly oriented powder (~10 μm) on a single crystal silicon substrate (27 mm diameter). We obtained semi-quantitative bulk mineralogy (including the total fraction of the sample comprising clay minerals) from diffraction patterns over 5–80° 2*θ*. The spectra were analyzed with the ICDD (International Centre for Diffraction Data) Powder Diffraction File-4+ database (http://www.icdd.com/products/pdf4.htm) and the reference intensity ratio after Snyder et al.^85^. To identify individual clay mineral species, analysis was performed from the 69–75° 2*θ* range, which yields distinct peaks for different clay minerals as a composite reflection arising from 060 and/or 33-1^86^. Using positions of quartz reflections as internal standards, all XRD peak positions were corrected for slight variations in sample height displacement error. Berthierine/chamosite, glauconite, Fe-smectite, Fe-illite, and kaolinite were the clay minerals identified and *f*_rw_sed_ was determined from this data (Fig. 5 and Supplementary Data Tables 4 and 5). Only cation-bearing clay minerals (berthierine/chamosite, glauconite, Fe-smectite, and Fe-illite) and quartz were included in determining *f*_rw_sed_. Kaolinite, a non-cation-bearing clay phase, was not incorporated as only cation-bearing clay phases consume alkalinity during formation. An increase in the abundance of berthierine/chamosite, Fe-smectite and Fe-illite were observed across the end-Permian mass extinction (Figs. 5 and 6 and Supplementary Data Tables 4 and 5). Correspondingly, the data reveal an increase in *f*_rw_sed_ and a decline in quartz abundance.

### Sediment mixing model

Mineralogical data derived from natural samples reflect a combination of both authigenic and detrital phases. This differs from the C–Si cycle output that reflects only the authigenic fraction. This difference can be addressed using a simple mixing equation (to allow for direct comparison of the authigenic *f*_rw_ model and sediment ratios) as follows:

7 $${f}_{{{{{{\rm{rw}}}}}}\_{{{{{\rm{s}}}}}}}={{{{{{\rm{M}}}}}}}_{{{{{{\rm{s}}}}}}-{{{{{\rm{a}}}}}}}.{f}_{{{{{{\rm{rw}}}}}}\_{{{{{\rm{s}}}}}}-{{{{{\rm{a}}}}}}}+{{{{{{\rm{M}}}}}}}_{{{{{{\rm{s}}}}}}-{{{{{\rm{d}}}}}}}.{f}_{{{{{{\rm{rw}}}}}}\_{{{{{\rm{s}}}}}}-{{{{{\rm{d}}}}}}}$$

where,

M_a_ = mass fractions of authigenic silica (SiO_2_) + authigenic clays

M_d_ = mass fractions of detrital silica (SiO_2_) + detrital clays

M_a_ + M_d_ = 1

*f*_rw___s_ = *f*_rw_ ratio expressed in sediment

*f*_rw___s-a_ = *f*_rw_ ratio of the authigenic fraction

*f*_rw___s-d_ = *f*_rw_ ratio of the detrital fraction.

Note that here authigenic silica refers to any non-detrital phase, including biogenic silica that forms in the water column. Using this simple expression and a Monte Carlo sampling approach, we estimate the changes in both the authigenic mass fraction (M_a_) and *f*_rw_s-a_ are required to drive the observed change in *f*_rw_s_. Foremost, we perform repeated calculations with the mixing equation (*n* = 500,000) by sampling uniformly for M_a_ and M_d_ between 0 and 1, *f*_rw_s-a_ between 0 and 1, and *f*_rw_s-d_ between 0 and 0.15 (to reproduce pre-extinction *f*_rw_s_ values of between 0–0.15 from both sections (Figs. 5 and 6)). Next, we filter the raw results for iterations that produce *f*_rw_s_ ranges (0–0.15 for pre-extinction, 0.15–0.55 for post extinction) expressed in the sedimentary record. The successful/filtered results are presented in Fig. 3 of the main text. We find that a *f*_rw_s-a_ of >0.2–0.65 is required to explain the observed *f*_rw_s_ values of 0.2–0.3 as derived from mineralogical analysis of both sections. Notably, an increase in temperature and acidity across the event would likely decrease (and not increase) the detrital mass fraction from pre- to post extinction.

## Supplementary information

Supplementary Information

## Data Availability

All data are available in the main text, Supplementary Materials, and files.

## Code availability

Code is available from the corresponding author on request.
