# Supplementary material for: Marine siliceous ecosystem decline led to sustained anomalous Early Triassic warmth
Source: Nat Commun. 2022 Jun 18;13:3509. doi: 10.1038/s41467-022-31128-3 (PMC9206662; doi:10.1038/s41467-022-31128-3)
Supplement: Supplementary file 1 — Supplementary Information [file 41467_2022_31128_MOESM1_ESM.pdf]

Supplementary Information: Marine siliceous ecosystem decline led to sustained anomalous Early Triassic warmth

Terry T. Isson[1], Shuang Zhang[2], Kimberly V. Lau[3], Sofia Rauzi[1], Nicholas J. Tosca[4], Donald E. Penman[5] and Noah J. Planavsky[6]

[1] Te Aka Mātuatua, University of Waikato (Tauranga), BOP, NZ
[2] Department of Oceanography, Texas A&M University, TX, USA
[3] Department of Geosciences and Earth and Environmental Systems Institute, Penn State University, PA, USA
[4] Department of Earth Sciences, University of Cambridge, Cambridge, UK
[5] Department of Geosciences, Utah State University, UT, USA
[6] Department of Geology and Geophysics, Yale University, CT, USA

Supplementary Figures 1-9

Supplementary Tables 1-5

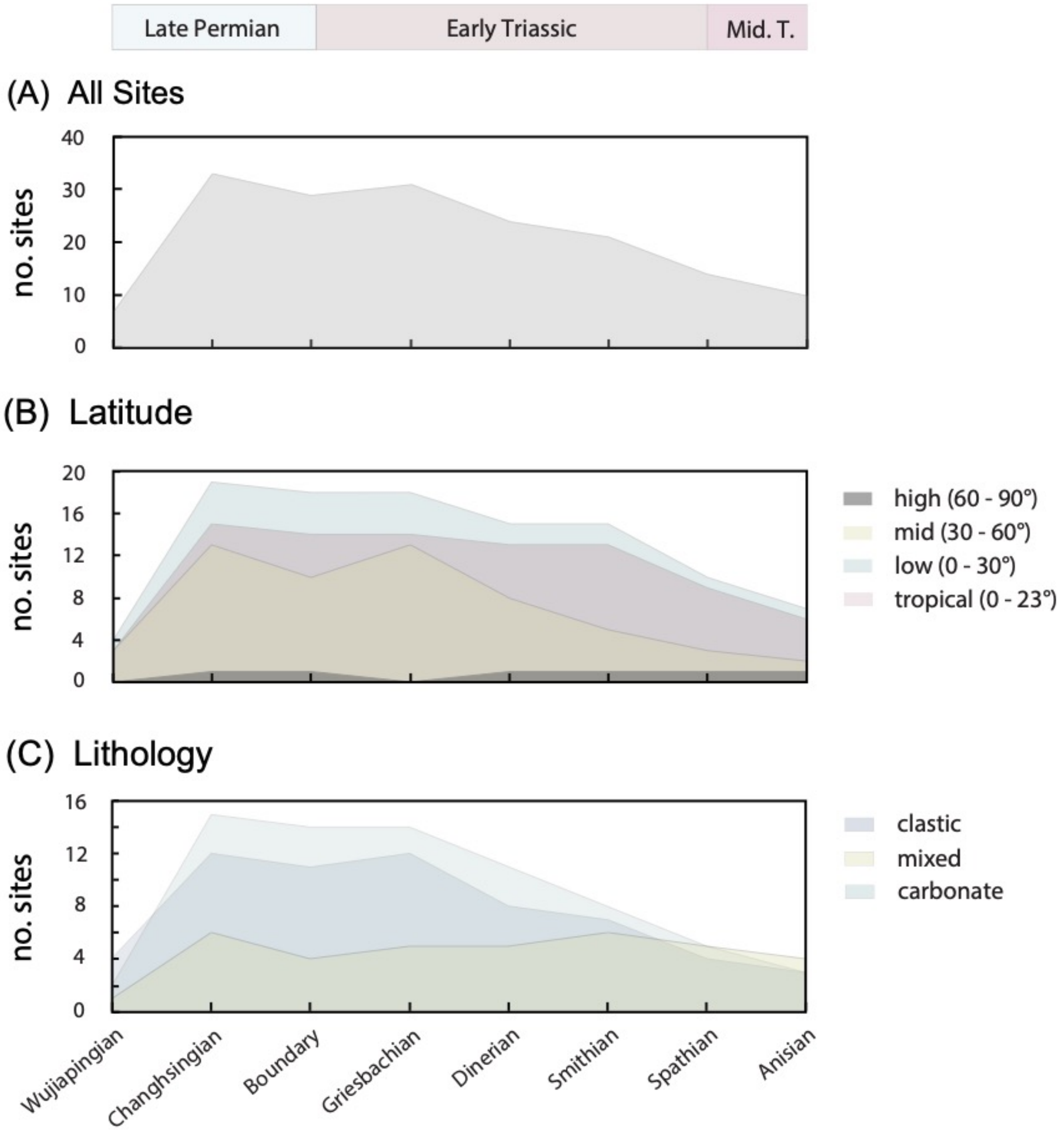

Supplementary Fig. 1 Marine sediment compilation indicating the number of sites available per time interval (substages except for the "Boundary") in (A) total, (B) by latitude and (C) by lithology.

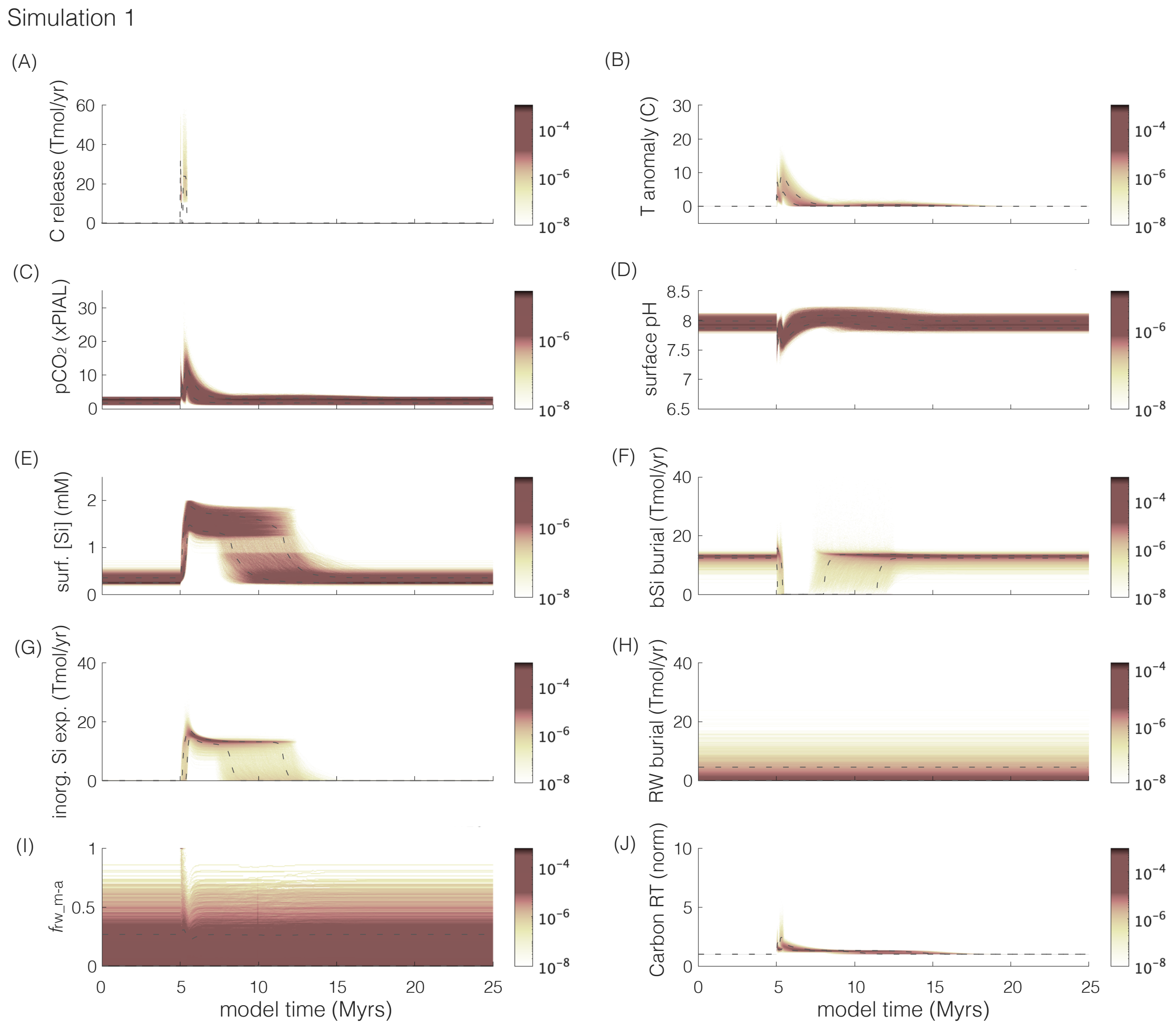

Supplementary Fig. 2 Simulation 1 (degassing + fixed carbon recycling) results (n=10,000). Color bar indicates frequency (normalized) of the results, 68% of values are within the dashed lines. The panels describe; (A) Carbon released from solid earth (volcanic) and sedimentary metamorphic degassing (Tmol/yr). Model time of 5 Myrs marks the initiation of volcanic carbon release and onset of extinction. Range of parameters explored: carbon release = 30,000–55,000 Pg; release duration = 0.8×10^5–0.24×10^6 years; and climate sensitivity = 2–5 °C (Supplementary Table 2-3); (B) temperature anomaly, (C) pCO_2 (times preindustrial atmospheric level (×PIAL)); (D) surface pH; (E) surface dissolved Si (mM); and (F) biogenic (G) inorganic and (H) authigenic clay silica burial fluxes (Tmol/yr); (I) frw; (J) residence time of carbon normalized to background value.

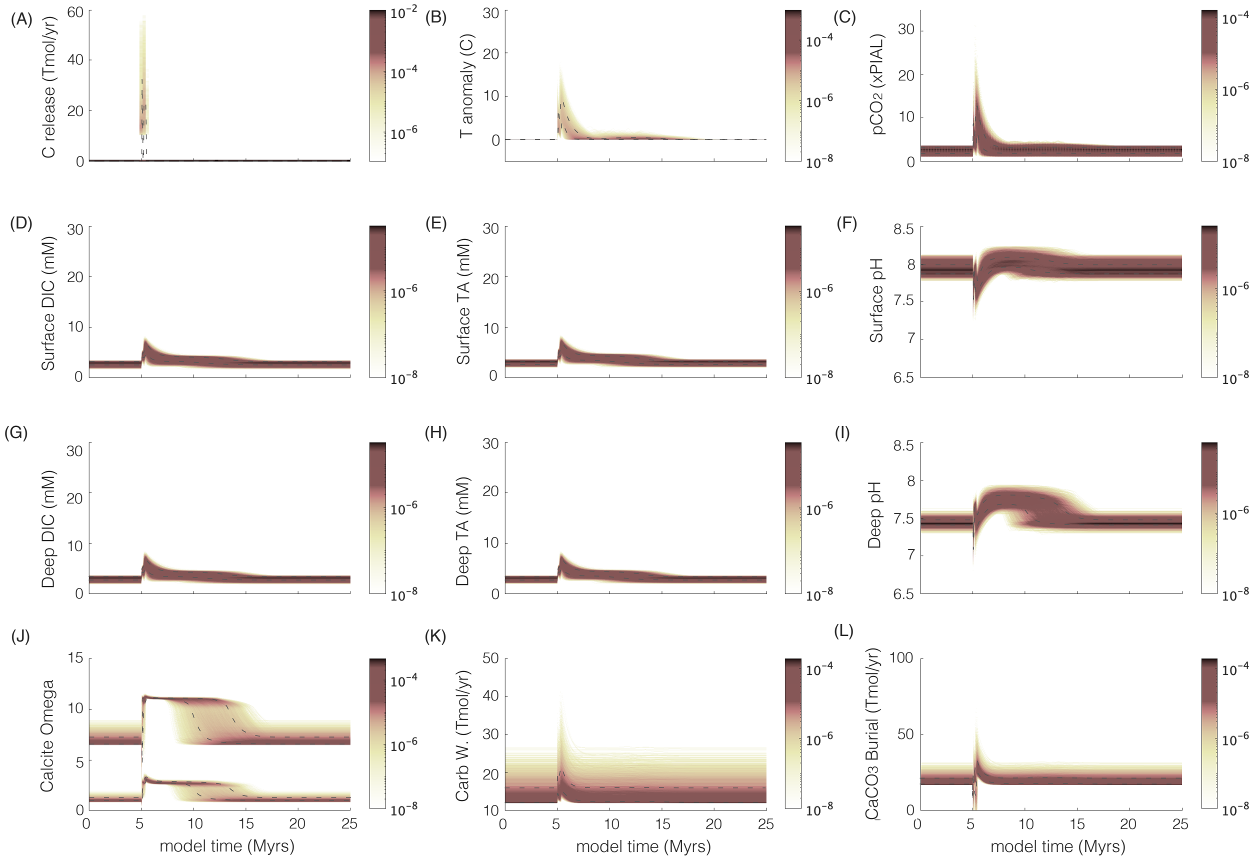

Supplementary Fig. 3 Unfiltered (raw) model results from Simulation 1 (carbon cycle) including (A) carbon release flux (Tmol/yr); (B) surface temperature anomaly (°C); (C) atmospheric pCO_2 (×PIAL); (D) surface DIC (mM); (E) surface TA (mM); (F) surface pH; (G) deep DIC (mM); (H) deep TA (mM); (I) deep pH; (J) calcite omega (top-surface, bottom-deep); (K) carbonate weathering (Tmol/yr); (L) total calcite burial (organic + inorganic) (Tmol/yr). Color bars indicates frequency (normalized) of the results, 68% of values are within the dashed lines.

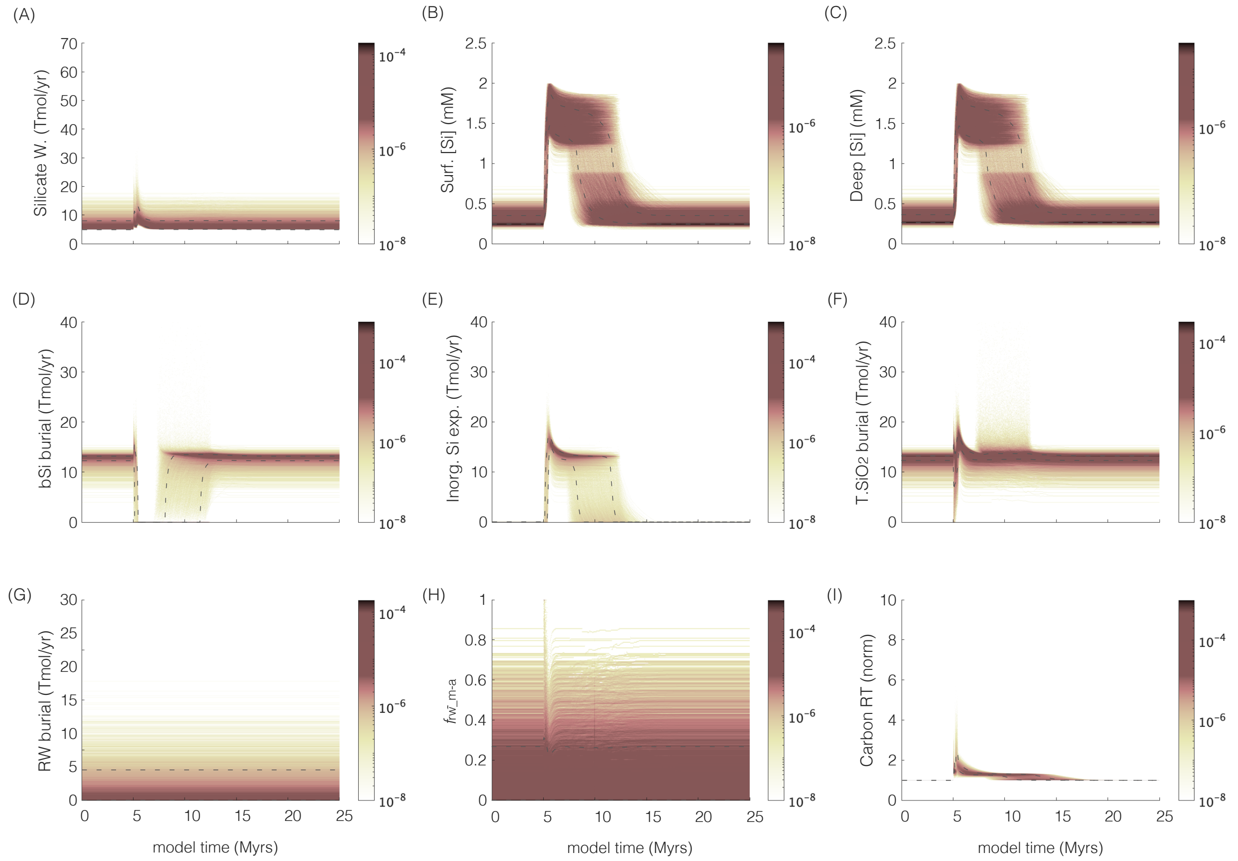

Supplementary Fig. 4 Unfiltered (raw) model results from Simulation 1 (silica cycle) including (A) silicate weathering (mol/yr); (B) surface dissolved silica (mM); (C) deep dissolved silica (mM); (D) biogenic silica burial (Tmol/yr); (E) inorganic silica burial (Tmol/yr); (F) total silica burial (Tmol/yr); (G) reverse weathering burial (Tmol/yr); (H) f_{rw} (fraction reverse weathering output of total silica output); (I) residence time of carbon (normalized to background) in the ocean-atmosphere system (years). Color bars indicates frequency (normalized) of the results, 68% of values are within the dashed lines.

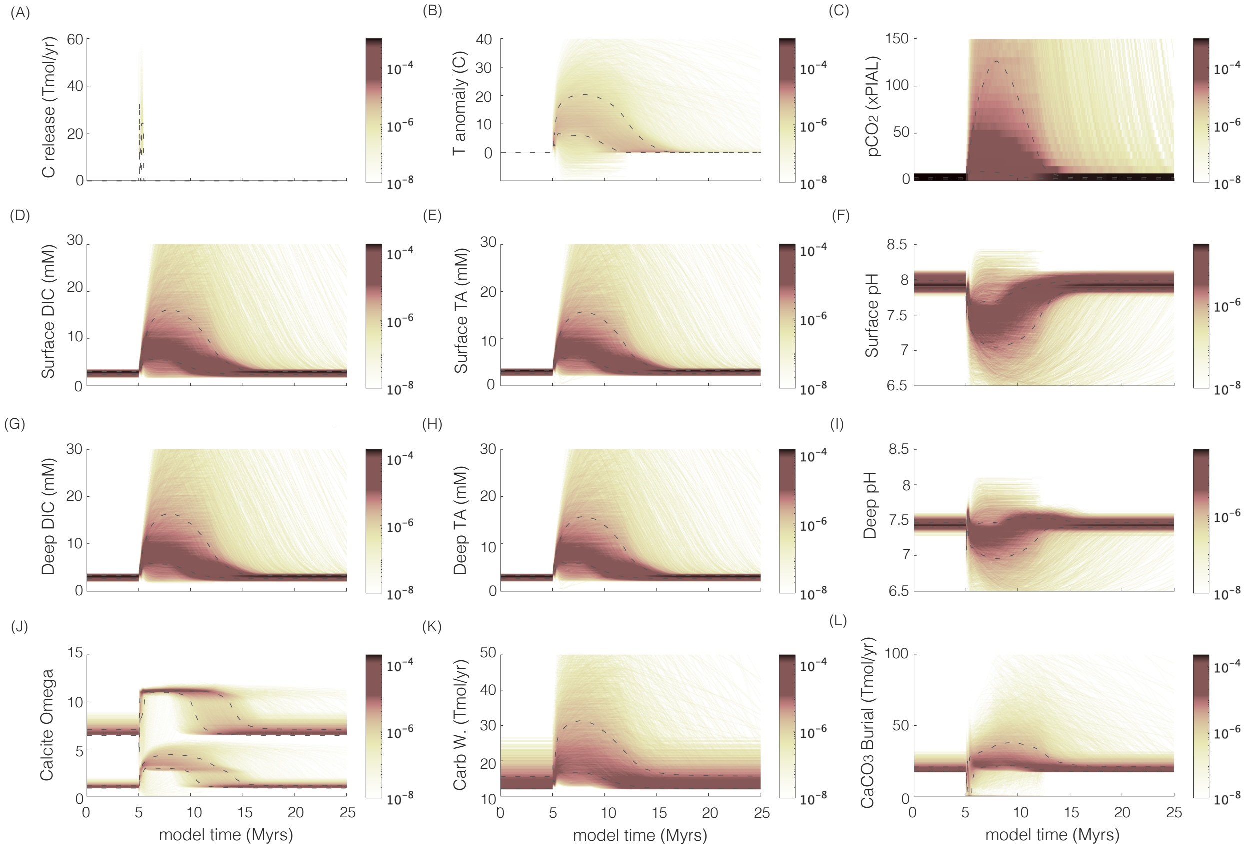

Supplementary Fig. 5 Unfiltered (raw) model results from Simulation 2 (carbon cycle) including (A) carbon release flux (Tmol/yr); (B) surface temperature anomaly (°C); (C) atmospheric pCO_2 (×PIAL); (D) surface DIC (mM); (E) surface TA (mM); (F) surface pH; (G) deep DIC (mM); (H) deep TA (mM); (I) deep pH; (J) calcite omega (top-surface, bottom-deep); (K) carbonate weathering (Tmol/yr); (L) total calcite burial (organic + inorganic) (Tmol/yr). Color bars indicates frequency (normalized) of the results, 68% of values are within the dashed lines.

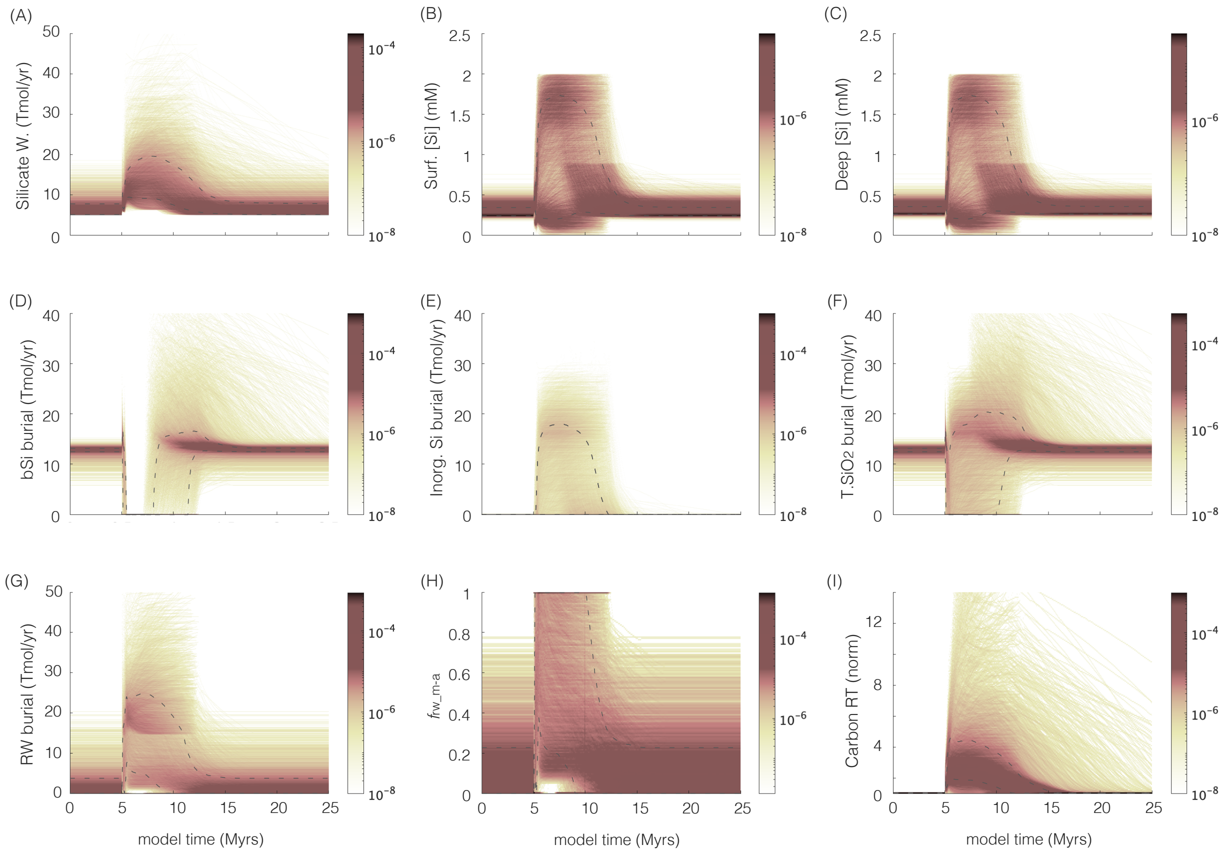

Supplementary Fig. 6 Unfiltered (raw) model results from Simulation 2 (silica cycle) including (A) silicate weathering (Tmol/yr); (B) surface dissolved silica (mM); (C) deep dissolved silica (mM); (D) biogenic silica burial (Tmol/yr); (E) inorganic silica burial (Tmol/yr); (F) total silica burial (Tmol/yr); (G) reverse weathering export (Tmol/yr); (H) f_{rw} (fraction reverse weathering export of total silica output); (I) residence time of carbon (normalized to background) in the ocean-atmosphere system (years). Color bars indicates frequency (normalized) of the results, 68% of values are within the dashed lines.

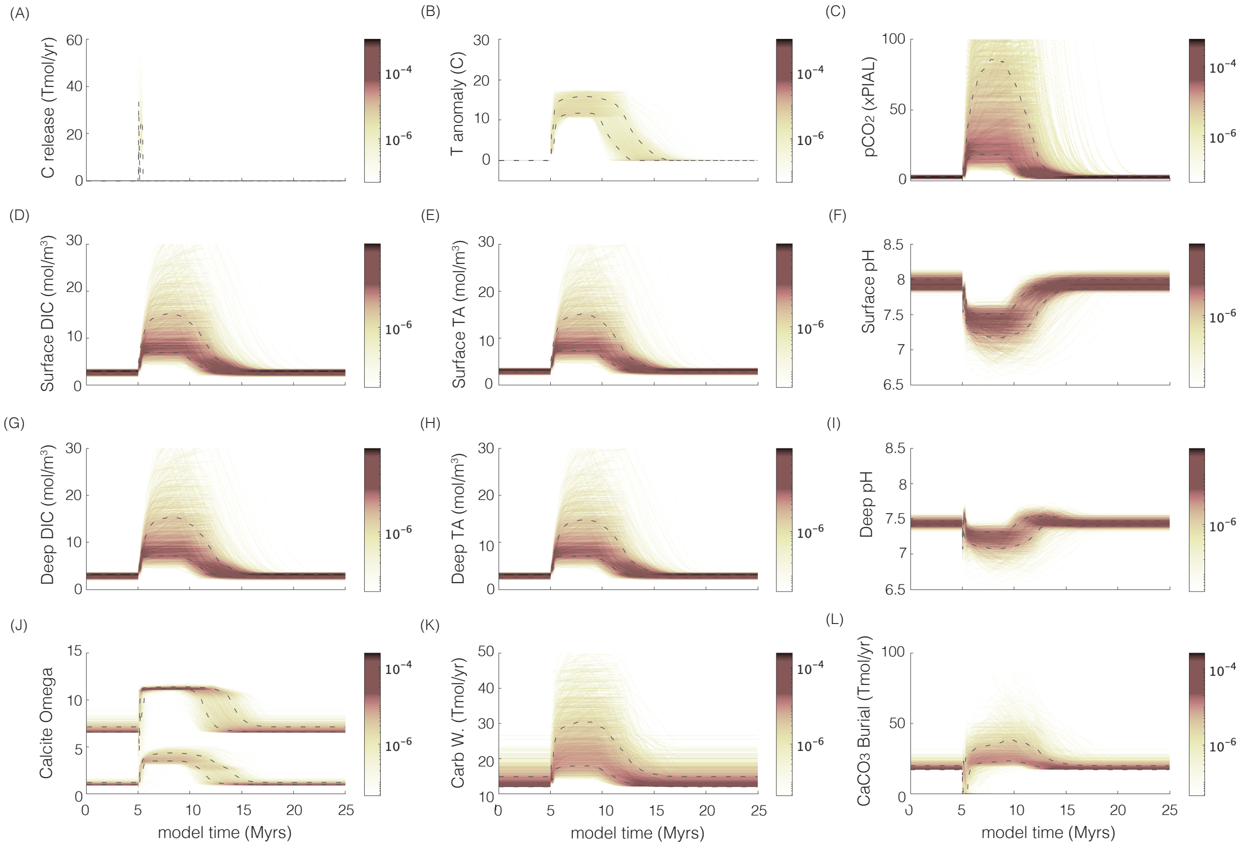

Supplementary Fig. 7 Filtered model results from Simulation 2 (carbon cycle) including (A) carbon release flux (Tmol/yr); (B) surface temperature anomaly (°C); (C) atmospheric pCO_2 (×PIAL); (D) surface DIC (mM); (E) surface TA (mM); (F) surface pH; (G) deep DIC (mM); (H) deep TA (mM); (I) deep pH; (J) calcite omega (top-surface, bottom-deep); (K) carbonate weathering (Tmol/yr); (L) total calcite burial (organic + inorganic) (Tmol/yr). Color bars indicates frequency (normalized) of the results, 68% of values are within the dashed lines.

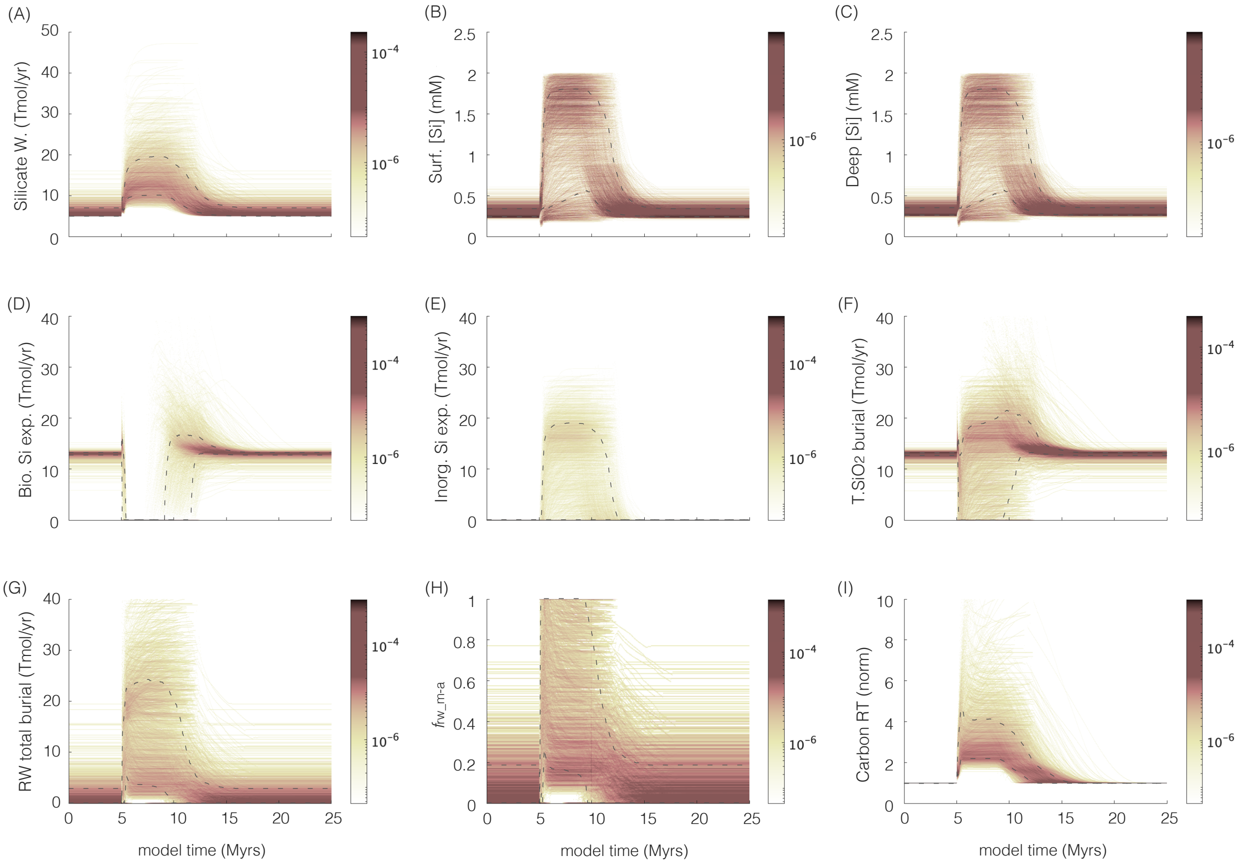

Supplementary Fig. 8 Filtered model results from Simulation 2 (silica cycle) including (A) silicate weathering (Tmol/yr); (B) surface dissolved silica (mM); (C) deep dissolved silica (mM); (D) biogenic silica burial (Tmol/yr); (E) inorganic silica burial (Tmol/yr); (F) total silica burial (Tmol/yr); (G) reverse weathering burial (Tmol/yr); (H) f_{rw} (fraction reverse weathering export of total silica output); (I) residence time of carbon (normalized to background) in the ocean-atmosphere system (years). Color bars indicates frequency (normalized) of the results, 68% of values are within the dashed lines.

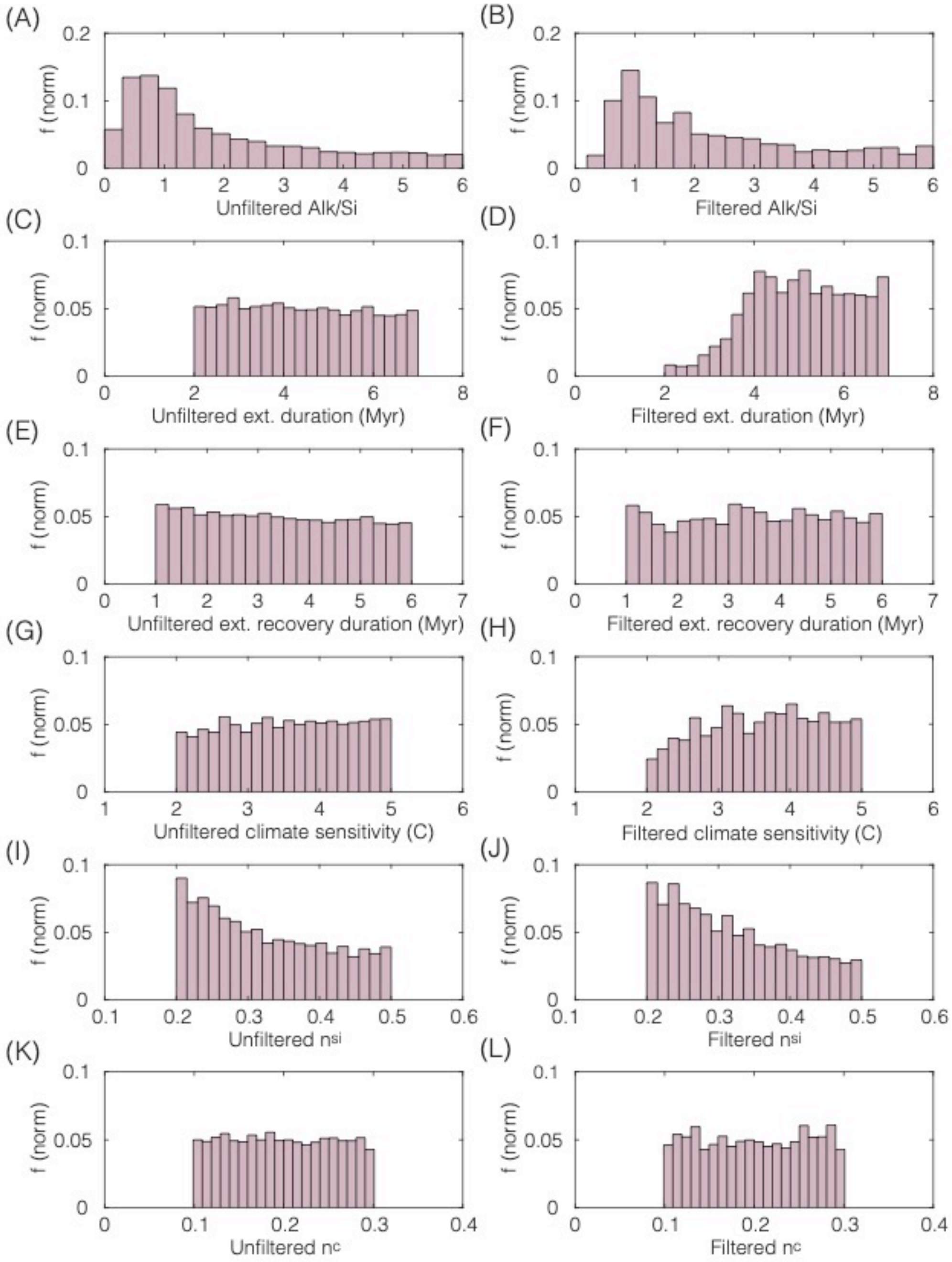

Supplementary Fig. 9 Distribution of results from Simulation 2 unfiltered/raw (left column) and filtered (based on temperature) / successful (right column) results as frequency (normalized). Distribution of (A-B) post-extinction Alk:Si, (C-D) extinction duration, (E-F) extinction recovery, (G-H) climate sensitivity, (I-J) n^{si} and (K-L) n^{c}.

Location	Paleo-latitude	Env.	LATE PERMIAN		EARLY TRIASSIC					M. TR.	Ref.
					Induan			Olenekian			
			Wuch.	Chang.	*boundary*	Gries.	Dienerian	Smithian	Spathian	Anisian	
Croatia	0	carbonate									3
Iran	-1	carbonate									4-7
Slovenia	2	carbonate									8
Vietnam	2	carbonate									9
Italy – Dolomites	2	carbonate									10-15
Italy – Sicily	2	mixed									16
Hungary	-3	carbonate									17
S. Tibet	5	carbonate									18
China – Nanpanjiang Basin	5	carbonate									19-22
China – Shangsi	11	clastic									16,23
Turkey – Taurides	-11	carbonate									5,24-27
Turkey – Bursa	-11	carbonate									16
W. USA – NV, Eastern CA	12	mixed									28
W. USA – Union Wash	12	mixed									27,29,30
Thailand	-15	clastic									31,32
China – Meishan	16	carbonate									33-35
Canada – BC	22	clastic									12,36,37
Japan – Akkamori	-24	clastic									38,39
Japan – Takachiho	28	carbonate									40
Greenland/Norway	29	clastic									41,42
UAE	-29	carbonate									43
Japan – Ubara	30	clastic									44,45
Japan – Gujo-Hachiman	30	clastic									44,46
Japan – Tenjinmaru	30	clastic									45
Japan – Ryugadake	30	clastic									45
Japan – Sasayama & Kinkazan	30	clastic									47
Oman – Wadi Maqam	-31	mixed									6,48
Oman – Wadi Aday	-31	carbonate									48
Oman – Wadi Sahtan	-31	carbonate									48
Oman – Wadi Wasit	-31	carbonate									49,50
Canada – Sverdrup	39	clastic									51-55
Norway – Spitsbergen	41	clastic									12,56,57
Norway – Spitsbergen	42	clastic									58
Pakistan	-43	mixed									59
India	-44	mixed									11,60
New Zealand	-72	mixed									61,62

Key		
Auth./diag. silica	Partial	Pervasive
Radiolarian silica	Partial	Pervasive
Sponge spicule silica	Partial	Pervasive
Radiolarian & sponge	Partial	Pervasive
No silica		
Not observed		

Supplementary Table 1 | Compilation of Late Permian to Middle Triassic marine sections including paleolatitude, lithology (clastic/carbonate) and the presence or absence of biogenic (radiolarian, sponge) and authigenic silica. 'Partial' denotes the observed presence of silica limited to one or a few fossil grains, clasts, or nodules, whereas 'pervasive' denotes the observed pervasive presence of silica on the bed-scale.

Supplementary Table 2 | Constant Model Parameters

Parameter	Symbol	Value	Unit	Ref.
Volcanic CO_2 degassing (background)	F_{vc}	5×10^{12}	mol yr^{-1}	[63-67]
$CaSiO_3$ weathering flux constant	F^0_{silw}	$= F_{vc}$	mol yr^{-1}	[63]
$CaCO_3$ weathering flux constant	F^0_{carbw}	12×10^{12}	mol yr^{-1}	[63]
SiO_2 weathering flux	F_{SiO2w}	$= F_{sillw} \times 0.19$	mol yr^{-1}	[68]
Dust Si input flux	F_{dust}	0.5×10^{12}	mol yr^{-1}	[68]
Hydrothermal Si input flux	F_{hyd}	1.7×10^{12}	mol yr^{-1}	[68]
Marine sediment silicate weathering	F_{mssw}	1.9×10^{12}	mol yr^{-1}	[68]
Groundwater Si input flux	F_{gw}	3.1×10^{12}	mol yr^{-1}	[68]
Organic biomass surface export	F_{org}	7.5×10^{14}	mol yr^{-1}	[69]
Organic biomass burial efficiency	$f_{\mathrm{org\_b}}$	0.031	-	[69]
Organic matter terrestrial weathering flux	F_{orgw}	$= F_{\mathrm{org}} \times f_{\mathrm{org\_b}}$	mol yr^{-1}	
Vertical mixing coefficient	V_{mix}	8	m yr^{-1}	[63,70,71]
Authigenic clay solubility constants	$[Si]_{\mathrm{o}}$	0.123	mM	[68,72]
(calibrated to reproduce modern flux)	$[H^+]_{\mathrm{o}}$	1.9×10^{-5}	mM	
Volcanic $\delta^{13}C$	$\delta^{13}C_{vc}$	- 4	‰	[63]
Terrestrial weathering $\delta^{13}C$	$\delta^{13}C_{w}$	+ 2	‰	[63]
Terrestrial organic matter $\delta^{13}C$	$\delta^{13}C_{orgw}$	- 23	‰	[66,73]
Carbonate-DIC $\delta^{13}C$ offset	$\Delta^{13}C_{carb\text{-}DIC}$	+ 0.5	‰	[74]
Organic biomass–DIC $\delta^{13}C$ fraction	$\Delta^{13}C_{org\text{-}DIC}$	- 27.7	‰	[63]
Seawater Ca	$[Ca]_{\mathrm{sw}}$	15	mM	[75,76]
Temperature filter (from mean)	-	temp: ± 4	°C	[77]
	-	age: ± 0.4	Myr	

Supplementary Table 3 | Monte Carlo Model Parameters

Parameter	Symbol	Value Lower	Value Upper	Unit	Ref.
Extinction duration	t_{ext}	2×10^{6}	7×10^{6}	years	[77-80]
Extinction recovery	t_{rec}	1×10^{6}	6×10^{6}	years	[77-80]
$CaSiO_3$ weathering exponent	n_{si}	0.2	0.5	-	[63,81]
$CaCO_3$ weathering exponent	n_{carb}	0.1	0.3	-	[63,81]
Authigenic clay Si exponent	r_{Si}	1	6 (1)	-	
Authigenic clay H^+ exponent	r_{H}	1	6 (2)	-	[64,72,82-92]
Authigenic clay Alk:Si consumption ratio	Alk:Si	0.17 (1)	6 (2)	-	
Inorganic silica solubility constant	∂	0.6	0.9	mol m^{-3}	[93,94]
Carbon injection total mass	M_{inj}	30000	55000	Pg	[95-97]
Carbon injection duration	d_{inj}	0.08×10^{6}	0.24×10^{6}	years	[95-97]
Carbon injection $\delta^{13}C$	$\delta^{13}C_w$	-40	-5	‰	[63,66,97]
Initial steady state temperature	T^0	17	19	°C	[98-100]
Weathering CO_2 constant	CO_2^c	60	800	ppm	[63]
Pre-extinction steady state CO_2	-	300	1000	ppm	[98,99,101]
Climate sensitivity	T_{sens}	2	5	°C	[102]

** pre-extinction values in parentheses*

Supplementary Table 4 | XRD results from Ubara (% rock)

depth (cm)	qtz (% rock)	pyr (% rock)	bth (% rock)	Fe-illite (% rock)	f_{rw_sed}
105	37	3	3	35	0.29
91	37	0	0	63	0.41
82	51	1	2	46	0.28
77	49	1	0	43	0.27
72	70	0	0	10	0.06
63	46	1	2	51	0.32
58	50	1	0	46	0.28
45	48	5	4	43	0.28
27	61	4	2	27	0.16
24	30	1	3	66	0.48
14	28	1	5	64	0.50
8	35	6	6	48	0.38
0	38	0	3	58	0.39
-1.5	27	1	3	69	0.52
-3	48	4	4	44	0.29
-4.5	34	1	5	60	0.43
-5	79	3	2	16	0.08
-7	62	5	2	31	0.18
-7.5	26	2	9	55	0.49
-9	36	3	12	49	0.39
-11	33	2	12	53	0.43
-13.5	35	3	8	52	0.40
-15	42	5	7	38	0.29
-17	25	1	7	61	0.52
-17.5	42	5	4	25	0.21
-18	56	1	4	35	0.22
-17.3	31	2	7	53	0.43
-20	35	1	4	29	0.27
-18.5	24	2	6	68	0.55
-19	34	1	2	32	0.29
-24	46	2	2	50	0.32
-21	43	1	2	54	0.35
-20	38	0	0	62	0.40
-23	44	2	2	52	0.33
-25	28		3	69	0.51
-26	66	2	0	26	0.14
-28	25	1	3	71	0.55
-32	40	0	1	55	0.37
-31	77	2	1	17	0.09
-33	79	1	1	17	0.08
-38	48	1	1	47	0.29
-34	56	4	2	38	0.22
-40	38	0	1	58	0.39
-54	42	2	1	52	0.34
-56	66	1	1	32	0.17
-64	81	0	0	17	0.08
-75	77	3	1	12	0.06
-101	77	0	0	19	0.09
-130	77	1	0	22	0.11

** Quartz (qtz); pyrite (pyr); berthierine (bth)*

Supplementary Table 5 | XRD results from Akkamori

**depth (cm)**	**qtz (% rock)**	**pyr (% rock)**	**cela (% rock)**	**bth (% rock)**	**Fe-smec (% rock)**	**glauc (% rock)**	**Fe-illite (% rock)**	**kaol (% rock)**	f_{rw_sed}
170.5	67	0	0	2.2	0.0	0	31	0	0.17
158.5	38	0	0	1.8	2.5	0	52	0	0.37
156.5	39	0	47	1.6	2.3	0	0	0	0.03
151.5	40	0	0	3.5	2.9	0	41	0	0.32
148.5	48	0	30	4.0	3.0	0	0	0	0.04
139.5	62	0	26	0.2	0.7	0	0	0	0.00
132.5	64	0	0	0.0	1.4	0	28	0	0.16
127	31	0	62	1.3	2.8	0	0	0	0.04
122.5	72	0	12	1.2	0.6	0	11	0	0.06
114.5	37	1	0	4.3	1.8	0	53	0	0.39
103.5	41	4	28	4.8	2.3	0	17	0	0.18
100	45	2	0	6.4	1.8	0	43	0	0.31
92.5	31	1	31	9.5	2.3	0	24	0	0.30
86.5	46	1	0	4.0	3.8	0	44	1	0.31
82	50	1	0	4.0	1.9	0	39	0	0.26
77	42	3	29	6.6	1.3	0	18	0	0.18
70	49	4	0	6.1	1.1	0	39	1	0.27
64	29	0	0	12.0	2.2	0	53	0	0.47
51.5	39	2	6	0.6	0.3	0	4	46	0.04
43.5	43	2	28	5.4	2.0	0	19	0	0.19
40	81	0	8	1.5	0.4	0	6	0	0.03
20	39	3	26	4.8	1.5	0	26	0	0.24
16.5	52	1	0	6.7	1.1	0	38	2	0.25
12	44	3	0	5.0	1.5	2	45	0	0.32
9.5	41	3	27	4.6	2.0	0	21	1	0.21
6	49	0	44	2.3	2.9	0	0	1	0.03
5	56	0	23	2.6	1.2	0	16	1	0.12
4	39	3	30	4.9	1.4	0	21	0	0.21
2.25	47	1	46	4.5	1.2	0	0	1	0.03
0.75	38	1	43	4.7	2.7	0	10	0	0.14
0	37	1	53	3.6	1.9	0	1	2	0.05
-2	63	1	33	1.8	1.4	0	0	0	0.01
-8	38	1	55	3.9	2.4	0	0	0	0.04
-9.8	41	0	55	0.8	2.4	1	0	0	0.04
-12	35	0	0	0.0	0.6	0	10	55	0.11
-14.5	46	0	48	3.1	1.4	0	0	1	0.03
-17	76	0	10	0.3	0.7	0	11	1	0.06
-20	30	1	36	4.3	2.0	0	27	0	0.30
-22	82	0	13	0.4	0.5	0	0	0	0.00
-26	39	2	28	4.5	1.9	0	17	0	0.19
-29	64	0	31	0.0	0.7	0	0	4	0.01
-32	28	3	63	4.3	2.0	0	0	0	0.06
-36	53	0	38	1.8	2.0	0	0	0	0.02
-40	28	0	0	2.5	3.0	0	50	1	0.45
-43	81	1	6	4.7	3.6	0	0	4	0.03
-49	30	0	63	2.6	3.0	0	0	2	0.05
-54	32	0	50	2.7	3.3	3	0	10	0.09
-59	40	0	18	2.1	6.9	7	0	26	0.14
-62	89	0	7	0.0	0.0	3	0	2	0.02
-65	63	0	37	0.0	0.0	0	0	0	0.00
-71	89	0	11	0.0	0.0	0	0	0	0.00
-74	29	0	66	0.0	4.1	0	0	0	0.05
-78	77	0	20	0.0	0.0	0	0	3	0.00
-89	30	0	66	0.0	0.0	0	0	0	0.00
-94	56	0	42	0.0	0.0	0	0	0	0.00
-100	43	0	35	0.0	0.0	0	0	0	0.00

** Quartz (qtz); pyrite (pyr); celadonite (cela); berthierine (bth); glauconite (glauc); kaolinite (kaol)*

Supplementary References

1 Takahashi, S., Nakada, R., Watanabe, Y. & Takahashi, Y. Iron-depleted pelagic water at the end-Permian mass extinction inferred from chemical species of iron and molybdenum in deep-sea sedimentary rocks. *Palaeogeography, palaeoclimatology, palaeoecology* **516**, 384-399 (2019).

2 Takahashi, S. *et al.* Progressive development of ocean anoxia in the end-Permian pelagic Panthalassa. *Global and Planetary Change* **207**, 103650 (2021).

3 Fio, K. *et al.* Stable isotope and trace element stratigraphy across the Permian–Triassic transition: A redefinition of the boundary in the Velebit Mountain, Croatia. *Chemical Geology* **278**, 38-57 (2010).

4 Sedlacek, A. R. *et al.* 87Sr/86Sr stratigraphy from the Early Triassic of Zal, Iran: Linking temperature to weathering rates and the tempo of ecosystem recovery. *Geology* **42**, 779-782 (2014).

5 Baud, A., Richoz, S. & Pruss, S. The lower Triassic anachronistic carbonate facies in space and time. *Global and Planetary Change* **55**, 81-89 (2007).

6 Richoz, S. *et al.* Permian–Triassic boundary interval in the Middle East (Iran and N. Oman): Progressive environmental change from detailed carbonate carbon isotope marine curve and sedimentary evolution. *Journal of Asian Earth Sciences* **39**, 236-253 (2010).

7 Horacek, M., Richoz, S., Brandner, R., Krystyn, L. & Spötl, C. Evidence for recurrent changes in Lower Triassic oceanic circulation of the Tethys: The δ13C record from marine sections in Iran. *Palaeogeography, Palaeoclimatology, Palaeoecology* **252**, 355-369 (2007).

8 Dolenec, T., Lojen, S. & Ramovš, A. The Permian–Triassic boundary in Western Slovenia (Idrijca Valley section): magnetostratigraphy, stable isotopes, and elemental variations. *Chemical Geology* **175**, 175-190 (2001).

9 Algeo, T. J., Ellwood, B., Nguyen, T. K. T., Rowe, H. & Maynard, J. B. The Permian–Triassic boundary at Nhi Tao, Vietnam: evidence for recurrent influx of sulfidic watermasses to a shallow-marine carbonate platform. *Palaeogeography, Palaeoclimatology, Palaeoecology* **252**, 304-327 (2007).

10 Groves, J. R., Rettori, R., Payne, J. L., Boyce, M. D. & Altiner, D. End-Permian mass extinction of lagenide foraminifers in the southern Alps (northern Italy). *Journal of Paleontology* **81**, 415-434 (2007).

11 Korte, C. *et al.* Massive volcanism at the Permian–Triassic boundary and its impact on the isotopic composition of the ocean and atmosphere. *Journal of Asian Earth Sciences* **37**, 293-311 (2010).

12 Wignall, P. B. & Twitchett, R. J. Extent, duration, and nature of the Permian-Triassic superanoxic event. *Special Papers-Geological Society of America*, 395-414 (2002).

13 Stefani, M., Furin, S. & Gianolla, P. The changing climate framework and depositional dynamics of Triassic carbonate platforms from the Dolomites. *Palaeogeography, Palaeoclimatology, Palaeoecology* **290**, 43-57 (2010).

14 Twitchett, R. J. Palaeoenvironments and faunal recovery after the end-Permian mass extinction. *Palaeogeography, Palaeoclimatology, Palaeoecology* **154**, 27-37 (1999).

15 Wignall, P. B. & Hallam, A. Anoxia as a cause of the Permian/Triassic mass extinction: facies evidence from northern Italy and the western United States. *Palaeogeography, Palaeoclimatology, Palaeoecology* **93**, 21-46 (1992).
16 Kozur, H. W. The systematic position of Pseudoertlispongus Lahm (Radiolaria) and description of some new Middle Triassic and Liassic radiolarian taxa. *Geol. Paläont. Mitt. Innsbruck* **4**, 287-297 (1996).
17 Hips, K. & Haas, J. Calcimicrobial stromatolites at the Permian–Triassic boundary in a western Tethyan section, Bükk Mountains, Hungary. *Sedimentary Geology* **185**, 239-253 (2006).
18 Wignall, P. B. & Newton, R. Contrasting deep-water records from the Upper Permian and Lower Triassic of South Tibet and British Columbia: evidence for a diachronous mass extinction. *Palaios* **18**, 153-167 (2003).
19 Lehrmann, D. J. *et al.* Permian and Triassic depositional history of the Yangtze platform and Great Bank of Guizhou in the Nanpanjiang basin of Guizhou and Guangxi, south China. *Albertiana* **33**, 149-168 (2005).
20 Lehrmann, D. J., Jiayong, W. & Enos, P. Controls on facies architecture of a large Triassic carbonate platform; the Great Bank of Guizhou, Nanpanjiang Basin, South China. *Journal of Sedimentary Research* **68**, 311-326 (1998).
21 Lehrmann, D. J. Early Triassic calcimicrobial mounds and biostromes of the Nanpanjiang basin, south China. *Geology* **27**, 359-362 (1999).
22 Meyer, K., Yu, M., Jost, A., Kelley, B. & Payne, J. δ13C evidence that high primary productivity delayed recovery from end-Permian mass extinction. *Earth and Planetary Science Letters* **302**, 378-384 (2011).
23 Wignall, P., Hallam, A., Xulong, L. & Fengqing, Y. Palaeoenvironmental changes across the Permian/Triassic boundary at Shangsi (N. Sichuan, China). *Historical Biology* **10**, 175-189 (1995).
24 Groves, J. R., Altiner, D. & Rettori, R. Extinction, survival, and recovery of lagenide foraminifers in the Permian–Triassic boundary interval, central Taurides, Turkey. *Journal of Paleontology* **79**, 1-38 (2005).
25 Kershaw, S. *et al.* Earliest Triassic microbialites in Çürük Dag, southern Turkey: composition, sequences and controls on formation. *Sedimentology* **58**, 739-755 (2011).
26 Loope, G. R., Kump, L. R. & Arthur, M. A. Shallow water redox conditions from the Permian–Triassic boundary microbialite: The rare earth element and iodine geochemistry of carbonates from Turkey and South China. *Chemical Geology* **351**, 195-208 (2013).
27 Pruss, S. B., Bottjer, D. J., Corsetti, F. A. & Baud, A. A global marine sedimentary response to the end-Permian mass extinction: examples from southern Turkey and the western United States. *Earth-science reviews* **78**, 193-206 (2006).
28 Sperling, E. A. & Ingle, J. C. A Permian–Triassic boundary section at Quinn River Crossing, northwestern Nevada, and implications for the cause of the Early Triassic chert gap on the western Pangean margin. *GSA Bulletin* **118**, 733-746 (2006).
29 Woods, A. D., Bottjer, D. J., Mutti, M. & Morrison, J. Lower Triassic large sea-floor carbonate cements: their origin and a mechanism for the prolonged biotic recovery from the end-Permian mass extinction. *Geology* **27**, 645-648 (1999).

30 Marenco, P. J., Griffin, J. M., Fraiser, M. L. & Clapham, M. E. Paleoecology and geochemistry of Early Triassic (Spathian) microbial mounds and implications for anoxia following the end-Permian mass extinction. *Geology* **40**, 715-718 (2012).
31 Sashida, K. Occurrence of Dienerian (Lower Triassic) radiolarians from the Phatthalung area of Peninsular Thailand and radiolarian biostratigraphy around the Permian/Triassic boundary. *News of Osaka Micropaleontologists* **11**, 59-70 (1998).
32 Sashida, K., Salyapongse, S. & Nakornsri, N. Latest Permian radiolarian fauna from Klaeng, eastern Thailand. *Micropaleontology* **46**, 245-263 (2000).
33 Burgess, S. D., Bowring, S. & Shen, S.-z. High-precision timeline for Earth's most severe extinction. *Proceedings of the National Academy of Sciences*, 201317692 (2014).
34 Cao, C. & Zheng, Q. Geological event sequences of the Permian-Triassic transition recorded in the microfacies in Meishan section. *Science in China Series D: Earth Sciences* **52**, 1529 (2009).
35 Jin, Y. *et al.* Pattern of marine mass extinction near the Permian-Triassic boundary in South China. *Science* **289**, 432-436 (2000).
36 Henderson, C. M. Uppermost Permian conodonts and the Permian-Triassic boundary in the western Canada sedimentary basin. *Bulletin of Canadian Petroleum Geology* **45**, 693-707 (1997).
37 Isozaki, Y. Permo-Triassic boundary superanoxia and stratified superocean: records from lost deep sea. *Science* **276**, 235-238 (1997).
38 Takahashi, S., Yamakita, S., Suzuki, N., Kaiho, K. & Ehiro, M. High organic carbon content and a decrease in radiolarians at the end of the Permian in a newly discovered continuous pelagic section: a coincidence? *Palaeogeography, Palaeoclimatology, Palaeoecology* **271**, 1-12 (2009).
39 Takahashi, S. *et al.* Sulfur isotope profiles in the pelagic Panthalassic deep sea during the Permian–Triassic transition. *Global and Planetary Change* **105**, 68-78 (2013).
40 Sano, H. & Nakashima, K. Lowermost Triassic (Griesbachian) microbial bindstone-cementstone facies, southwest Japan. *Facies* **36**, 1-24 (1997).
41 Georgiev, S. *et al.* Hot acidic Late Permian seas stifle life in record time. *Earth and Planetary Science Letters* **310**, 389-400 (2011).
42 Bugge, T. *et al.* Upper Permian as a new play model on the mid-Norwegian continental shelf: Investigated by shallow stratigraphic drilling. *AAPG bulletin* **86**, 107-127 (2002).
43 Clarkson, M. *et al.* Ocean acidification and the Permo-Triassic mass extinction. *Science* **348**, 229-232 (2015).
44 Algeo, T. J. *et al.* Spatial variation in sediment fluxes, redox conditions, and productivity in the Permian–Triassic Panthalassic Ocean. *Palaeogeography, Palaeoclimatology, Palaeoecology* **308**, 65-83 (2011).
45 Kakuwa, Y. Evaluation of palaeo-oxygenation of the ocean bottom across the Permian–Triassic boundary. *Global and Planetary Change* **63**, 40-56 (2008).
46 Algeo, T. J. *et al.* Changes in productivity and redox conditions in the Panthalassic Ocean during the latest Permian. *Geology* **38**, 187-190 (2010).
47 Kato, Y., Nakao, K. & Isozaki, Y. Geochemistry of Late Permian to Early Triassic pelagic cherts from southwest Japan: implications for an oceanic redox change. *Chemical Geology* **182**, 15-34 (2002).

48 Weidlich, O. & Bernecker, M. in *Third EAGE Workshop on Arabian Plate Geology.* cp-271-00032 (European Association of Geoscientists & Engineers).
49 Twitchett, R., Krystyn, L., Baud, A., Wheeley, J. & Richoz, S. Rapid marine recovery after the end-Permian mass-extinction event in the absence of marine anoxia. *Geology* **32**, 805-808 (2004).
50 Wheeley, J. & Twitchett, R. Palaeoecological significance of a new Griesbachian (Early Triassic) gastropod assemblage from Oman. *Lethaia* **38**, 37-45 (2005).
51 Beauchamp, B. & Baud, A. Growth and demise of Permian biogenic chert along northwest Pangea: evidence for end-Permian collapse of thermohaline circulation. *Palaeogeography, Palaeoclimatology, Palaeoecology* **184**, 37-63 (2002).
52 Algeo, T. *et al.* Evidence for a diachronous Late Permian marine crisis from the Canadian Arctic region. *GSA Bulletin* **124**, 1424-1448 (2012).
53 Beauchamp, B. & Grasby, S. E. Permian lysocline shoaling and ocean acidification along NW Pangea led to carbonate eradication and chert expansion. *Palaeogeography, Palaeoclimatology, Palaeoecology* **350**, 73-90 (2012).
54 Grasby, S., Beauchamp, B., Embry, A. & Sanei, H. Recurrent Early Triassic ocean anoxia. *Geology* **41**, 175-178 (2013).
55 Grasby, S. E., Chen, Z. & Dewing, K. Formation water geochemistry of the Sverdrup Basin: Implications for hydrocarbon development in the High Arctic. *Applied Geochemistry* **27**, 1623-1632 (2012).
56 Wignall, P. B. & Twitchett, R. J. Oceanic anoxia and the end Permian mass extinction. *Science* **272**, 1155-1158 (1996).
57 Wignall, P., Morante, R. & Newton, R. The Permo-Triassic transition in Spitsbergen: δ 13 C org chemostratigraphy, Fe and S geochemistry, facies, fauna and trace fossils. *Geological Magazine* **135**, 47-62 (1998).
58 Foster, W. J., Danise, S. & Twitchett, R. J. A silicified Early Triassic marine assemblage from Svalbard. *Journal of Systematic Palaeontology* **15**, 851-877 (2017).
59 Hermann, E. *et al.* Organic matter and palaeoenvironmental signals during the Early Triassic biotic recovery: The Salt Range and Surghar Range records. *Sedimentary Geology* **234**, 19-41 (2011).
60 Algeo, T. J. *et al.* Sequencing events across the Permian–Triassic boundary, Guryul Ravine (Kashmir, India). *Palaeogeography, Palaeoclimatology, Palaeoecology* **252**, 328-346 (2007).
61 Takemura, A. *et al.* Triassic radiolarians from the ocean-floor sequence of the Waipapa Terrane at Arrow Rocks, Northland, New Zealand. *New Zealand Journal of Geology and Geophysics* **45**, 289-296 (2002).
62 Hori, R., Campbell, J. & Grant-Mackie, J. Triassic Radiolaria from Kaka Point Structural Belt, Otago, New Zealand. *Journal of the Royal Society of New Zealand* **33**, 39-55 (2003).
63 Zeebe, R. LOSCAR: Long-term ocean-atmosphere-sediment carbon cycle reservoir model v2. 0.4. *Geoscientific Model Development* **5**, 149-166 (2012).
64 Isson, T. *et al.* Evolution of the Global Carbon Cycle and Climate Regulation on Earth. *Global Biogeochemical Cycles* (2020).
65 Stewart, E. *et al.* (Mineralogical Society of America, 2019).
66 Kump, L. R. & Arthur, M. A. Interpreting carbon-isotope excursions: carbonates and organic matter. *Chemical Geology* **161**, 181-198 (1999).

67 Coogan, L. & Gillis, K. The average Phanerozoic CO2 degassing flux estimated from the O-isotopic composition of seawater. *Earth and Planetary Science Letters* **536**, 116151 (2020).
68 Tréguer, P. J. *et al.* Reviews and syntheses: The biogeochemical cycle of silicon in the modern ocean. *Biogeosciences Discussions*, 1-43 (2021).
69 Dunne, J. P., Sarmiento, J. L. & Gnanadesikan, A. A synthesis of global particle export from the surface ocean and cycling through the ocean interior and on the seafloor. *Global Biogeochemical Cycles* **21** (2007).
70 Yool, A. & Tyrrell, T. Role of diatoms in regulating the ocean's silicon cycle. *Global Biogeochemical Cycles* **17** (2003).
71 Broecker, W. & Peng, T. Tracers in the Sea, 690 pp. *Lamont-Doherty Geological Observatory, Palisades, NY* (1982).
72 Gaboreau, S., Gailhanou, H., Blanc, P., Vieillard, P. & Made, B. Clay mineral solubility from aqueous equilibrium: Assessment of the measured thermodynamic properties. *Applied Geochemistry* **113**, 104465 (2020).
73 Garcia, A. K., Cavanaugh, C. M. & Kacar, B. The curious consistency of carbon biosignatures over billions of years of Earth-life coevolution. *The ISME Journal*, 1-12 (2021).
74 Mackensen, A. & Schmiedl, G. Stable carbon isotopes in paleoceanography: atmosphere, oceans, and sediments. *Earth-Science Reviews* **197**, 102893 (2019).
75 Horita, J., Zimmermann, H. & Holland, H. D. Chemical evolution of seawater during the Phanerozoic: Implications from the record of marine evaporites. *Geochimica et Cosmochimica Acta* **66**, 3733-3756 (2002).
76 Turchyn, A. V. & DePaolo, D. J. Seawater chemistry through Phanerozoic time. *Annual Review of Earth and Planetary Sciences* **47**, 197-224 (2019).
77 Sun, Y. *et al.* Lethally hot temperatures during the Early Triassic greenhouse. *Science* **338**, 366-370 (2012).
78 Fraiser, M. L. & Bottjer, D. J. Elevated atmospheric CO2 and the delayed biotic recovery from the end-Permian mass extinction. *Palaeogeography, Palaeoclimatology, Palaeoecology* **252**, 164-175 (2007).
79 Chen, Z.-Q. & Benton, M. J. The timing and pattern of biotic recovery following the end-Permian mass extinction. *Nature Geoscience* **5**, 375 (2012).
80 Foster, W. J. & Twitchett, R. J. Functional diversity of marine ecosystems after the Late Permian mass extinction event. (2013).
81 Penman, D. E., Rugenstein, J. K. C., Ibarra, D. E. & Winnick, M. J. Silicate weathering as a feedback and forcing in Earth's climate and carbon cycle. *Earth-Science Reviews*, 103298 (2020).
82 Mackenzie, F., Ristvet, B., Thorstenson, D., Lerman, A. & Leeper, R. Reverse weathering and chemical mass balance in a coastal environment. (1981).
83 Mackenzie, F. T. & Kump, L. R. Reverse weathering, clay mineral formation, and oceanic element cycles. *Science* **270**, 586 (1995).
84 Mackenzie, F. T. & Garrels, R. M. Chemical mass balance between rivers and oceans. *American Journal of Science* **264**, 507-525 (1966).
85 Hazen, R. M. *et al.* Clay mineral evolution. *American Mineralogist* **98**, 2007-2029 (2013).
86 Tosca, N. & Masterson, A. Chemical controls on incipient Mg-silicate crystallization at 25 C: Implications for early and late diagenesis. *Clay Minerals* **49**, 165-194 (2014).

87 Tosca, N. J., Guggenheim, S. & Pufahl, P. K. An authigenic origin for Precambrian greenalite: Implications for iron formation and the chemistry of ancient seawater. *Geological Society of America Bulletin* **128**, 511-530 (2016).
88 Ehlert, C. *et al.* Stable silicon isotope signatures of marine pore waters–Biogenic opal dissolution versus authigenic clay mineral formation. *Geochimica et Cosmochimica Acta* **191**, 102-117 (2016).
89 Michalopoulos, P. & Aller, R. C. Rapid clay mineral formation of Amazon delta sediments: Reverse weathering and oceanic elemental cycles. *Science* **270**, 614 (1995).
90 Michalopoulos, P. & Aller, R. C. Early diagenesis of biogenic silica in the Amazon delta: alteration, authigenic clay formation, and storage. *Geochimica et Cosmochimica Acta* **68**, 1061-1085 (2004).
91 Michalopoulos, P., Aller, R. C. & Reeder, R. J. Conversion of diatoms to clays during early diagenesis in tropical, continental shelf muds. *Geology* **28**, 1095-1098 (2000).
92 Gainey, S. R. *et al.* Clay mineral formation under oxidized conditions and implications for paleoenvironments and organic preservation on Mars. *Nature communications* **8**, 1-7 (2017).
93 Siever, R. Silica solubility, 0-200 C., and the diagenesis of siliceous sediments. *The Journal of Geology* **70**, 127-150 (1962).
94 Siever, R. The silica cycle in the Precambrian. *Geochimica et Cosmochimica Acta* **56**, 3265-3272 (1992).
95 Sobolev, S. V. *et al.* Linking mantle plumes, large igneous provinces and environmental catastrophes. *Nature* **477**, 312 (2011).
96 Svensen, H. *et al.* Siberian gas venting and the end-Permian environmental crisis. *Earth and Planetary Science Letters* **277**, 490-500 (2009).
97 Cui, Y., Li, M., van Soelen, E. E., Peterse, F. & Kürschner, W. M. Massive and rapid predominantly volcanic CO2 emission during the end-Permian mass extinction. *Proceedings of the National Academy of Sciences* **118** (2021).
98 Jurikova, H. *et al.* Permian–Triassic mass extinction pulses driven by major marine carbon cycle perturbations. *Nature Geoscience*, 1-6 (2020).
99 Cui, Y. & Kump, L. R. Global warming and the end-Permian extinction event: Proxy and modeling perspectives. *Earth-Science Reviews* **149**, 5-22 (2015).
100 Song, H. *et al.* Thresholds of temperature change for mass extinctions. *Nature communications* **12**, 1-8 (2021).
101 Royer, D. L., Berner, R. A., Montañez, I. P., Tabor, N. J. & Beerling, D. J. CO2 as a primary driver of phanerozoic climate. *GSA Today* **14**, 4-10 (2004).
102 Roe, G. H. & Baker, M. B. Why is climate sensitivity so unpredictable? *Science* **318**, 629-632 (2007).
